# Computational Evaluation of Carbazole–Chalcone Urea and Thiourea Derivatives Against EGFR: Molecular Docking, Pharmacokinetic Profiling, Target Prediction, and Molecular Dynamics Simulation

**DOI:** 10.3390/molecules31193372

**Published:** 2026-09-23

**Authors:** Arleta Rifati-Nixha, Mustafa Arslan, Miribane Dërmaku-Sopjani, Aida Buza-Hapçiu

**Affiliations:** 1 Chemistry Department, Faculty of Mathematical & Natural Sciences, Universiteti i Prishtinës, 10000 Prishtina, Kosovo; arleta.rifati@uni-pr.edu (A.R.-N.);; 2 Chemistry Department, Faculty of Arts and Sciences, Sakarya University, 54187 Sakarya, Turkey

**Keywords:** carbazole derivatives, EGFR, molecular docking, molecular dynamics, SwissADME, SwissTargetPrediction, drug-likeness

## Abstract

A series of previously synthesized carbazole-based urea and thiourea derivatives (**5a**–**5i**) was subjected to an integrated computational investigation of their predicted interactions with epidermal growth factor receptor (EGFR) (PDB ID: 1M17) and their pharmacokinetic and dynamic behavior. Molecular docking yielded scores of −9.0 to −13.5 kcal/mol, with **5f** showing the most favorable value (−13.5 kcal/mol). Redocking of the co-crystallized ligand AQ4 yielded an RMSD of 1.65 Å, supporting the docking protocol. SwissADME analysis revealed high lipophilicity, low predicted gastrointestinal absorption, and poor aqueous solubility across the series. Compound **5f** showed a Consensus LogP of 7.05, two Lipinski violations, a bioavailability score of 0.17, and predicted P-glycoprotein substrate behavior. SwissTargetPrediction identified EGFR among the predicted targets of **5f**, providing complementary computational rationale for its investigation. Three independent 100 ns molecular dynamics simulations of the EGFR–5f complex showed limited protein backbone deviations (mean RMSD: 0.217–0.282 nm), maintained overall compactness (mean Rg: 2.005–2.040 nm), and continued protein–ligand association despite replicate-dependent conformational sampling. Overall, **5f** was computationally prioritized based on its favorable predicted EGFR interactions and sustained association over the investigated simulation timescale, while its physicochemical and pharmacokinetic limitations indicate the need for further structural optimization and experimental validation.

## 1. Introduction

Chalcones constitute a structurally versatile class of α,β-unsaturated ketones that has attracted considerable interest in medicinal chemistry because of the broad range of biological activities associated with this scaffold, including antimicrobial, antioxidant, anti-inflammatory, anticancer, and enzyme-inhibitory effects [1,2,3,4]. Their synthetic accessibility and considerable structural diversity allow systematic modification of both aromatic rings and the α,β-unsaturated carbonyl framework, making chalcone derivatives valuable scaffolds for investigating structure-dependent biological properties [5,6]. Accordingly, structural modification and molecular hybridization of the chalcone framework have been extensively explored as strategies for modulating biological activity, molecular recognition, and physicochemical properties relevant to the development of new bioactive molecules [5,6,7,8].

Among nitrogen-containing heterocyclic systems, **carbazole** represents another privileged scaffold of considerable relevance to medicinal chemistry. Carbazole-containing compounds have been associated with antimicrobial, antioxidant, anticancer, and enzyme-inhibitory activities, while their extended aromatic system provides opportunities for hydrophobic, π-mediated, and other non-covalent interactions with biological macromolecules [9,10,11,12]. The combination of carbazole and chalcone-derived structural features within a single molecular framework therefore represents a rational molecular-hybridization strategy for expanding chemical diversity and exploring complementary interaction patterns with biological targets [9,10,11]. Such hybrid structures can be further diversified through the incorporation of pharmacophoric functionalities, including **urea and thiourea groups**, which provide hydrogen-bond donor and acceptor sites and may contribute to ligand–protein molecular recognition.

The molecular hybridization strategy underlying the investigated series is schematically illustrated in Figure 1, highlighting the integration of the carbazole scaffold, chalcone-derived framework, and urea/thiourea functionality within a single molecular architecture.

The **epidermal growth factor receptor (EGFR)** is a receptor tyrosine kinase involved in signaling pathways governing cellular proliferation, survival, differentiation, and other processes relevant to tumor development and progression. Dysregulation of EGFR signaling has been implicated in several malignancies, making its kinase domain a well-established molecular target in anticancer drug discovery. Importantly, the rationale for investigating EGFR in the present study is supported by increasing evidence that **chalcone-containing hybrid structures can interact with and inhibit EGFR**. Xanthine–chalcone hybrids have been synthesized and experimentally evaluated as EGFR inhibitors and anticancer agents, with molecular modeling further supporting their interaction with the EGFR binding site [13]. Similarly, thienoquinoline–chalcone derivatives have demonstrated antiproliferative activity together with direct EGFR kinase inhibition, with docking analysis indicating accommodation of the chalcone-containing structures within the EGFR binding region [14]. In addition, 1,3,4-oxadiazole–chalcone hybrids have been experimentally shown to inhibit EGFR and related signaling targets [15]. More recently, **chalcone–urea derivatives** have also been reported as potent EGFR inhibitors, providing particularly relevant precedent for investigating urea-containing chalcone scaffolds against this kinase [16].

These findings provide a structural and bibliographic basis for exploring EGFR recognition by the **carbazole–chalcone urea and thiourea derivatives** investigated in the present work. Nevertheless, EGFR has not been experimentally established as a target of compounds **5a**–**5i**. Their previously reported inhibitory activity against polyphenol oxidase (PPO) therefore does not constitute the basis for assigning EGFR as their biological target. Rather, EGFR was selected here as an **exploratory molecular target** based on literature evidence for EGFR recognition and inhibition by structurally related chalcone-containing hybrid systems, together with the capacity of the present scaffold to provide multiple aromatic, hydrophobic, and hydrogen-bonding interaction features. This distinction is important because the present computational investigation is intended to generate a testable target-oriented hypothesis rather than to establish EGFR inhibition in the absence of direct biochemical evidence.

Computational approaches have become increasingly important in medicinal chemistry as complementary tools for examining molecular recognition and prioritizing compounds before more resource-intensive experimental investigations. **Molecular docking** is widely used to predict plausible ligand orientations within protein binding sites and to estimate relative docking scores within a defined computational framework [17,18,19,20,21,22]. Beyond compound ranking, analysis of predicted binding poses can provide structural information on hydrogen-bonding, hydrophobic, π-related, halogen, and other non-covalent interactions potentially contributing to ligand–protein recognition. The reliability of a docking protocol can be further assessed through validation procedures such as redocking of a co-crystallized ligand and comparison of the predicted and experimentally observed poses. Integrated docking strategies have consequently been employed in synthetic and computational investigations aimed at characterizing and prioritizing potential bioactive compounds [23,24,25,26,27,28,29,30,31,32,33]. Recent EGFR-oriented computational studies have similarly combined docking with complementary molecular modeling approaches to investigate ligand recognition and prioritize potential EGFR-interacting compounds [34,35].

Docking predictions alone, however, provide a predominantly static representation of protein–ligand recognition and do not account for physicochemical and pharmacokinetic properties that may substantially influence the further development of a compound. **In silico** pharmacokinetic and drug-likeness profiling therefore provides a complementary level of assessment during early-stage compound evaluation [36,37,38,39,40,41]. The **SwissADME** platform enables prediction of relevant molecular and physicochemical descriptors, including molecular weight, lipophilicity, topological polar surface area, aqueous solubility, gastrointestinal absorption, blood–brain barrier permeation, and compliance with established drug-likeness criteria [42]. Its graphical tools, including the **Bioavailability Radar** and **BOILED-Egg**, further facilitate interpretation of physicochemical space, predicted passive gastrointestinal absorption, BBB permeation, and P-glycoprotein-related **behavior** [42]. **Such** parameters are important when assessing whether favorable target-oriented molecular recognition is accompanied by physicochemical characteristics compatible with further compound development [36,37,38,39].

**Complementary target-prediction approaches** provide an additional perspective by identifying potential protein targets on the basis of molecular similarity to compounds with known biological activity. **SwissTargetPrediction** enables ligand-based exploration of potential macromolecular targets and can therefore be used to examine whether a target investigated by structure-based docking is independently represented within the predicted target spectrum [43]. In the context of the present study, this approach is particularly relevant for evaluating whether **EGFR emerges among the predicted targets of compound 5f**, thereby providing an independent computational line of evidence complementary to structure-based docking. Nevertheless, such predictions remain exploratory and cannot substitute for direct biochemical or cellular validation of target engagement.

Beyond static docking models, **molecular dynamics (MD) simulations** provide a time-dependent framework for investigating the structural behavior of protein–ligand complexes under simulated conditions. Analysis of complementary descriptors such as root-mean-square deviation (RMSD), root-mean-square fluctuation (RMSF), radius of gyration (Rg), protein–ligand distance, intermolecular contacts, and hydrogen bonding can provide information on structural fluctuations, protein compactness, ligand reorientation, and the persistence of protein–ligand association over the simulated timescale. Recent computational investigations have increasingly integrated molecular docking, pharmacokinetic prediction, and MD simulations to provide a more comprehensive characterization of prioritized compounds and protein–ligand complexes [34,35,44,45,46,47]. Such integrated computational approaches have also been applied specifically to EGFR-oriented investigations, supporting their utility for examining ligand recognition, dynamic behavior, and predicted pharmacokinetic properties prior to experimental validation [34,35,47]. Such analyses are particularly useful when interpreted as complementary computational evidence rather than as substitutes for experimental validation.

In our previous study, we reported the synthesis, structural characterization, theoretical investigation, and biological evaluation of a series of **carbazole-substituted chalcone urea and thiourea derivatives (5a**–**5i)** as polyphenol oxidase (PPO) inhibitors, with several derivatives exhibiting inhibitory activity [48]. Although the synthetic and PPO-related properties of these compounds have already been described, their potential molecular recognition within the EGFR kinase binding site and their predicted physicochemical, pharmacokinetic, and target profiles have not been comprehensively investigated. The present work therefore extends the characterization of this previously synthesized series toward a distinct, literature-supported molecular target while explicitly recognizing that the predicted EGFR interaction requires subsequent experimental validation.

Accordingly, the present study aimed to provide an integrated **in silico** characterization of compounds **5a**–**5i**, with particular emphasis on their predicted interaction with the **EGFR tyrosine kinase (PDB ID: 1M17)**. Molecular docking, together with redocking of the co-crystallized ligand, was employed to evaluate predicted binding orientations and interaction patterns within the EGFR binding site. **SwissADME** and **SwissTargetPrediction** analyses were subsequently used to characterize physicochemical and drug-likeness properties and to explore potential biological targets. Based on its comparative docking performance, compound **5f** was prioritized for more detailed evaluation using the Bioavailability Radar and BOILED-Egg models and for **three independent 100 ns molecular dynamics simulations** of the EGFR–5f complex. By integrating docking, pharmacokinetic profiling, target prediction, and extended replicate-based dynamic analysis, this study sought to identify both favorable molecular-recognition features and physicochemical liabilities of the investigated scaffold and to establish a rational basis for subsequent structural optimization and experimental evaluation.

## 2. Materials and Methods

### 2.1. Chemistry

The carbazole–chalcone urea and thiourea derivatives (**5a**–**5i**) investigated in the present study were previously synthesized and fully characterized according to our reported synthetic methodology [48]. The structural assignments of the R and X substituents for compounds **5a**–**5i** are summarized in Table 1. Their molecular structures were established using **FT-IR, ^1^H NMR, ^13^C NMR spectroscopy, and elemental analysis**. Since the synthetic procedures and spectroscopic characterization data have already been published, these experimental details are not repeated in the present work. Instead, the previously characterized molecular structures were used as the basis for the subsequent **molecular docking, in silico pharmacokinetic and drug-likeness profiling, target prediction, and molecular dynamics simulations**. For clarity and completeness, the previously reported synthetic route leading to compounds **5a**–**5i** is reproduced in Scheme 1 [48].

### 2.2. Compounds Investigated in the Computational Study

The carbazole–chalcone urea and thiourea derivatives **5a**–**5i** were included in the present computational investigation. These compounds had previously been evaluated for polyphenol oxidase (PPO) inhibitory activity [48]; however, their inclusion in the present study was **not based on the magnitude of the reported PPO inhibition**, since PPO activity does not provide a direct basis for predicting interaction with EGFR. Instead, the complete series was investigated to enable a consistent comparison of their predicted molecular recognition within the **EGFR tyrosine kinase binding site**, together with their physicochemical, pharmacokinetic, drug-likeness, and target-prediction profiles.

The previously reported PPO data were considered only as part of the biological background of the compound series and were not used to infer EGFR-related activity. The complete chemical structures of compounds **5a**–**5i** are presented in **Section 3** **(Figure 2)** for reference.

### 2.3. Molecular Docking Studies

Molecular docking studies were performed to evaluate the predicted binding modes, docking scores, and interaction patterns of compounds **5a**–**5i** toward the **epidermal growth factor receptor (EGFR) tyrosine kinase** (PDB ID: **1M17**). The crystal structure of EGFR was retrieved from the **Protein Data Bank (PDB)**. Prior to docking, the receptor structure was prepared by removing the co-crystallized ligand and crystallographic water molecules. Polar hydrogen atoms were added, and Kollman partial charges were assigned using **AutoDockTools (version 1.5.7)** [31].

The ligand structures were prepared and energy-minimized using **Avogadro version 2.0.0** with the **MMFF94 force field**. The optimized structures were subsequently imported into **PyRx** in PDB format and converted to **PDBQT** format for docking calculations. No separate protonation-state or tautomer-enumeration procedure was applied; therefore, the protonation and tautomeric forms represented by the optimized input structures were retained during ligand preparation, and no alternative tautomeric forms were explicitly generated.

Molecular docking calculations for compounds **5a**–**5i** were carried out using **AutoDock Vina** [49], as implemented in the **PyRx (version 0.8)** virtual-screening platform [50]. The docking search space was centered on the EGFR ligand-binding region, with grid-center coordinates of **X = 23.5415, Y = 9.8480, and Z = 59.4052 Å**. The grid-box dimensions were set to **25 × 25 × 25 Å^3^**, providing coverage of the ligand-binding pocket. The **exhaustiveness parameter was set to 8**. A single docking run was performed for each ligand, generating **nine binding poses per compound**. The resulting Vina docking scores were expressed in **kcal/mol**. For subsequent protein–ligand interaction analysis, the pose with the most favorable predicted docking score was selected; when poses exhibited comparable docking scores, their orientation and plausible interaction pattern within the EGFR active site were additionally considered.

To validate the docking protocol, the co-crystallized ligand **AQ4** was extracted from the EGFR crystal structure and independently re-docked into the binding site. For the re-docking procedure, the search space was centered at **X = 22.014, Y = 0.253, and Z = 52.794 Å**, with grid-box dimensions of **22.5 × 18.0 × 18.0 Å^3^**. AutoDock Vina was applied with an **exhaustiveness value of 16**, a maximum of **20 generated binding modes**, and an **energy range of 4 kcal/mol**. The generated poses were compared with the experimentally observed crystallographic orientation using the **root-mean-square deviation (RMSD)**. An RMSD value below **2.0 Å** was considered indicative of satisfactory reproduction of the crystallographic binding pose [51]. The selected re-docked pose (**Mode 3**) yielded an RMSD of **1.65 Å** relative to the crystallographic AQ4 pose and was therefore used to support validation of the docking protocol.

The selected protein–ligand complexes were subsequently inspected using **Discovery Studio Visualizer (version 25.1.0.24284)** to characterize the principal predicted non-covalent interactions within the EGFR binding site, including conventional hydrogen bonds, hydrophobic interactions, π-related interactions, halogen interactions, and van der Waals contacts.

### 2.4. In Silico Pharmacokinetic, Drug-likeness, and Target-Prediction Analyses

The physicochemical, drug-likeness, and pharmacokinetic properties of compounds **5a**–**5i** were evaluated in silico using the **SwissADME** web server [42]. The molecular structures of the investigated compounds were used as input to calculate relevant physicochemical descriptors, including molecular weight (MW), lipophilicity (LogP), topological polar surface area (TPSA), hydrogen-bond donors (HBDs), hydrogen-bond acceptors (HBAs), and the number of rotatable bonds (RBs). Predicted gastrointestinal (GI) absorption, blood–brain barrier (BBB) permeation, compliance with **Lipinski’s rule of five** [36,39], bioavailability score, and aqueous solubility according to the ESOL model (LogS) were also evaluated. These descriptors were collectively considered to characterize the predicted drug-likeness and pharmacokinetic profiles of the investigated derivatives.

For compound **5f**, which showed the most favorable docking score within the investigated series, the **Bioavailability Radar** and **BOILED-Egg** models implemented in SwissADME were additionally examined [42]. The Bioavailability Radar was used to visualize the physicochemical profile of **5f** in terms of lipophilicity, molecular size, polarity, solubility, flexibility, and saturation, whereas the BOILED-Egg model was used to assess predicted passive gastrointestinal absorption, blood–brain barrier permeation, and P-glycoprotein (P-gp) substrate status [42].

Potential biological targets were further explored using the **SwissTargetPrediction** web server [43]. Particular attention was given to compound **5f** to determine whether its predicted target profile provided additional computational support for the EGFR-directed docking investigation. SwissTargetPrediction analysis was also performed for the remaining derivatives **5a**–**5i**, and the corresponding extended target-prediction results are included in the Supplementary Material. Target predictions were interpreted as exploratory in silico estimates based on structural similarity to compounds with known biological activity [43] and were not considered evidence of direct target engagement.

### 2.5. Molecular Dynamics Simulation

Molecular dynamics (MD) simulations were performed using **GROMACS (version 2026.3)** [52] to investigate the dynamic behavior of compound **5f** in complex with the epidermal growth factor receptor **(EGFR; PDB ID: 1M17).** The protein was described using the **CHARMM27 force field** [53], whereas the topology and force-field parameters of compound **5f** were generated using **SwissParam** [54] for compatibility with the CHARMM-based force field. The protein–ligand complex was solvated using the **TIP3P water model** [55] under periodic boundary conditions. The solvated system contained **13,586 water molecules, 42 Na^+^ ions, and 43 Cl^−^ ions**.

Prior to equilibration, the system was subjected to energy minimization using the **steepest-descent algorithm** for a maximum of **50,000 steps**, with an energy-minimization tolerance (emtol) of **1000 kJ mol^−1^ nm^−1^** and an initial minimization step size (emstep) of **0.01 nm**. A **Verlet cutoff scheme** was employed, with short-range electrostatic and van der Waals cutoff distances of **1.0 nm**. Long-range electrostatic interactions were treated using the **particle mesh Ewald (PME)** method [56], and periodic boundary conditions were applied in all three spatial dimensions.

Following energy minimization, the system was equilibrated sequentially under **NVT and NPT ensembles**, with position restraints applied during both equilibration stages. The NVT equilibration was conducted for **100 ps** using a **2 fs time step** at a reference temperature of **300 K**. Temperature was controlled using the **velocity-rescaling (V-rescale) thermostat** [57] with a coupling constant (*τ*t) of **0.1 ps**. The protein–ligand complex and the water/ion environment were coupled separately to the temperature bath. The equilibrated NVT system was subsequently subjected to **100 ps of NPT equilibration** at **300 K and 1 bar**. Pressure was controlled isotropically using the **Berendsen barostat** [58], with a pressure-coupling constant (*τ*p) of **2.0 ps** and a compressibility of **4.5 × 10^−5^ bar^−1^**.

To improve sampling and assess the reproducibility of the observed structural behavior, **three independent 100 ns production MD simulations** were performed for the EGFR–5f complex. Each replicate comprised **50,000,000 integration steps** using a **2 fs time step**. The independent trajectories were generated using separately initialized equilibration trajectories before production simulation. During production, the temperature was maintained at **300 K** using the V-rescale thermostat [57], while pressure was maintained at **1 bar** using an isotropic **Parrinello–Rahman barostat** [59], with *τ*p = **2.0 ps** and a compressibility of **4.5 × 10^−5^ bar^−1^**. Covalent bonds involving hydrogen atoms were constrained using the **LINCS algorithm** [60]. Long-range electrostatic interactions were treated with PME [56], while short-range Coulomb and van der Waals interactions employed a **1.0 nm cutoff**. Periodic boundary conditions were maintained in all three dimensions. Trajectory coordinates were saved every **10 ps** for subsequent analysis.

The three MD trajectories were analyzed using complementary structural and protein–ligand interaction descriptors. **Protein backbone RMSD** was calculated following least-squares fitting to the corresponding starting protein backbone, while **ligand RMSD** was evaluated after backbone fitting to assess positional and conformational changes in compound **5f** relative to its initial binding pose. Residue-level protein flexibility was characterized using **Cα RMSF**, and global protein compactness was evaluated using the **radius of gyration (Rg)**. Protein–ligand association was further examined through the **minimum protein–ligand distance, atomic contact analysis, and hydrogen-bond analysis**. Protein–ligand atomic contacts were defined using a distance cutoff of **0.35 nm**. Hydrogen bonds were evaluated using the geometric criteria implemented in GROMACS, including a donor–acceptor distance cutoff of **0.35 nm**. These descriptors were evaluated across all three independent **100 ns trajectories** to characterize replicate-dependent conformational behavior, protein compactness, ligand reorientation, and protein–ligand association over the simulated timescale.

## 3. Results and Discussion

### 3.1. Previously Reported Structural Characterization

The chemical structures of compounds **5a**–**5i** were previously confirmed by **FT-IR, ^1^H NMR, ^13^C NMR spectroscopy, and elemental analysis**. The spectroscopic and analytical data were consistent with the proposed structures and have been reported in detail in our previous publication [48]. Therefore, these characterization data are not discussed further in the present study, which focuses on the **computational evaluation of the previously synthesized derivatives**.

The chemical structures of compounds **5a**–**5i** investigated in the present computational study are shown in Figure 2 for reference.

### 3.2. Molecular Docking Results

The molecular docking analysis revealed predicted docking scores ranging from **−9.0 to −13.5 kcal/mol** for compounds **5a**–**5i**, indicating substantial variation in their predicted interaction with the EGFR tyrosine kinase binding site. Among the investigated derivatives, compound **5f**, bearing a **3-CF_3_** substituent, exhibited the most favorable docking score (**−13.5 kcal/mol**), whereas compound **5b** showed the least favorable value (**−9.0 kcal/mol**). The complete docking results are summarized in Table 2.

For docking-protocol validation, the co-crystallized ligand **AQ4** was independently re-docked using the dedicated re-docking parameters described in Section 2.3. The selected validation pose (**Mode 3**) yielded a docking score of **−6.8 kcal/mol** and an RMSD of **1.65 Å** relative to the crystallographic orientation. The AQ4 docking score is included in Table 1 as a contextual reference to the validation experiment and was not used as a direct quantitative measure of relative experimental binding affinity. Accordingly, the docking scores of compounds **5a**–**5i** were interpreted comparatively within the investigated series.

Comparison across the investigated series revealed substantial differences in predicted docking scores among compounds bearing different substituents. Although compound **5f (3-CF_3_)** exhibited the most favorable predicted score, the results do not indicate a simple or monotonic relationship between an individual substituent and docking performance. This is illustrated by compound **5b (3-F)**, which showed the least favorable score in the series despite also bearing a substituent at the meta position. Therefore, the favorable docking behavior of compound **5f** is more appropriately interpreted as reflecting the combined influence of its overall molecular geometry, steric complementarity, hydrophobic character, and electrostatic interactions within the EGFR binding pocket rather than an isolated effect of the **3-CF_3_** substituent.

Overall, the observed variation in docking scores likely reflects the combined influence of multiple structural and physicochemical factors, including ligand geometry, steric complementarity, hydrophobicity, and electrostatic interactions, rather than a straightforward substituent-dependent trend. Consequently, the docking data were not interpreted as establishing an experimentally validated structure–activity relationship. Instead, they were used as a comparative computational criterion for prioritizing compounds for more detailed evaluation. Compound **5f** was selected for subsequent pharmacokinetic profiling, target-prediction analysis, and molecular dynamics simulation because it exhibited the most favorable predicted docking score within the investigated series.

#### 3.2.1. Docking Protocol Validation by Redocking

To assess the ability of the docking workflow to reproduce the experimentally observed binding orientation, the co-crystallized ligand **AQ4** was independently re-docked into the EGFR binding site (PDB ID: **1M17**) using the dedicated re-docking parameters described in Section 2.3. The generated poses were compared with the crystallographic orientation of AQ4 by calculating the **root-mean-square deviation (RMSD)**.

Among the generated poses, **Mode 3** showed the closest agreement with the crystallographic pose, with an RMSD of **1.65 Å** and a docking score of **−6.8 kcal/mol**. Although **Mode 1** exhibited a slightly more favorable docking score (**−7.0 kcal/mol**), its substantially higher RMSD value (**5.93 Å**) indicated poor reproduction of the experimentally observed ligand orientation. Therefore, **Mode 3** was selected as the representative re-docked pose for validation of the docking workflow.

The RMSD value of **1.65 Å**, which is below the commonly applied **2.0 Å criterion for successful re-docking** [51], indicates satisfactory reproduction of the crystallographic AQ4 orientation and supports the suitability of the adopted docking workflow for the subsequent computational analysis. The superposition of the crystallographic and selected re-docked poses is presented in **Figure 3**.

#### 3.2.2. Predicted Binding Mode and Interaction Profile of Compound **5f**

Following validation of the docking protocol, the predicted binding modes of compounds **5a**–**5i** within the **EGFR binding site** were examined. Among the investigated derivatives, compound **5f** exhibited the most favorable docking score (**−13.5 kcal/mol**) and was therefore selected for a more detailed analysis of its predicted binding orientation and protein–ligand interaction pattern. The three-dimensional representation of compound **5f** within the EGFR binding pocket is presented in **Figure 4**, while the corresponding two-dimensional interaction map is shown in **Figure 5**.

Analysis of the two-dimensional interaction map revealed a diverse network of predicted non-covalent contacts between compound **5f** and residues within the EGFR binding pocket. Conventional hydrogen-bond interactions were observed with **Asp105** and **Met98**, while the trifluoromethyl moiety participated in fluorine-mediated halogen contacts with **Arg146**. The ligand was additionally involved in π-related interactions, including a **π–sigma interaction with Leu149** and a **π–anion interaction with Asp160**. Several **alkyl and π–alkyl interactions**, together with additional van der Waals contacts involving surrounding residues, further contributed to the predicted interaction network within the binding pocket.

Collectively, the predicted combination of hydrogen-bonding, hydrophobic, π-related, halogen, and van der Waals contacts is **consistent with the favorable docking score observed for compound 5f**. Nevertheless, these interactions represent predictions derived from the docking model and should not be interpreted as evidence of experimentally confirmed binding or EGFR inhibition. The favorable docking performance of **5f** therefore served as the basis for its selection for subsequent **SwissADME, SwissTargetPrediction, and molecular dynamics analyses**.

### 3.3. ADMET, Drug-likeness, and Pharmacokinetic Analyses

The pharmacokinetic and drug-likeness properties of compounds **5a**–**5i** were evaluated using in silico prediction tools, and the calculated descriptors are summarized in **Table 3**.

The molecular weights ranged from **459.54 to 601.50 g/mol**, with compound **5d** exhibiting the highest molecular weight due to the presence of the iodine substituent, whereas compound **5a** showed the lowest value. The predicted **Consensus LogP** values ranged from **4.44 to 7.05**, indicating pronounced lipophilic character across the investigated series. Compound **5f** exhibited the highest predicted lipophilicity, whereas compound **5i** showed the lowest Consensus LogP value.

The calculated **TPSA** values ranged from **63.13 to 123.97 Å^2^**. The highest TPSA was observed for compound **5c**, followed by **5i**, consistent with the presence of additional polar functionalities in these nitro-substituted derivatives. All compounds contained **two hydrogen-bond donor groups**, whereas the number of hydrogen-bond acceptors ranged from **two to five**. In addition, the derivatives contained **eight or nine rotatable bonds**, indicating a relatively high degree of conformational flexibility.

The predicted pharmacokinetic parameters indicated **low gastrointestinal absorption** and **no blood–brain barrier permeation** for all derivatives. Compound **5f** was additionally predicted to be a **P-glycoprotein substrate**, whereas the remaining derivatives were predicted to be non-substrates. Compounds **5a**, **5b**, **5c**, **5e**, and **5i** showed **one Lipinski rule violation** and a predicted **bioavailability score of 0.55**, whereas compounds **5d** and **5f**–**5h** showed **two Lipinski rule violations** and a lower bioavailability score of **0.17**.

The predicted **ESOL LogS** values ranged from **−6.45 to −8.81**, indicating low predicted aqueous solubility across the series. Compound **5a** showed the least negative ESOL LogS value (**−6.45**), whereas compound **5d** exhibited the most negative value (**−8.81**). Importantly, **no PAINS alerts** were identified for any of the investigated derivatives. In contrast, one or two **Brenk structural alerts** were predicted, depending on the compound. The synthetic accessibility scores ranged from **3.36 to 3.68**, suggesting broadly comparable predicted synthetic accessibility across the series.

Overall, the in silico analysis revealed a **mixed pharmacokinetic profile** for the investigated derivatives. Although the compounds showed no predicted BBB permeation and no PAINS alerts, their relatively high lipophilicity, low predicted gastrointestinal absorption, and poor predicted aqueous solubility represent potential limitations. In particular, compound **5f**, despite exhibiting the most favorable docking score within the investigated series (**−13.5 kcal/mol**), showed comparatively high lipophilicity (**Consensus LogP = 7.05**), two Lipinski rule violations, a predicted bioavailability score of **0.17**, and P-glycoprotein substrate behavior. Thus, its **favorable predicted docking performance toward EGFR should be considered alongside these pharmacokinetic limitations**, which suggest that further structural optimization may be required.

#### Bioavailability Radar, BOILED-Egg, and SwissTargetPrediction Analyses

To provide a more detailed assessment of the predicted physicochemical, pharmacokinetic, and target profile of compound **5f**, which exhibited the most favorable docking score within the investigated series, additional analyses were performed using the **SwissADME** and **SwissTargetPrediction** web servers. The Bioavailability Radar, BOILED-Egg model, and SwissTargetPrediction results obtained for compound **5f** are presented in **Figure 6**.

The **Bioavailability Radar** provides a graphical representation of six physicochemical properties relevant to oral drug likeness, namely lipophilicity (LIPO), molecular size (SIZE), polarity (POLAR), solubility (INSOLU), flexibility (FLEX), and saturation (INSATU). The optimal physicochemical space is represented by the pink region, whereas the red profile corresponds to the calculated properties of compound **5f**. As shown in **Figure 6A**, several descriptors of compound **5f** fall within or close to the recommended physicochemical range, whereas deviations are evident, particularly for lipophilicity and solubility. These observations are consistent with its high **Consensus LogP value of 7.05** and poor predicted aqueous solubility (**ESOL LogS = −7.78**) reported in Table 2. The pronounced lipophilic character of **5f** is consistent with the contribution of its hydrophobic aromatic framework and trifluoromethyl substituent.

The **BOILED-Egg** model was subsequently examined to assess the predicted passive gastrointestinal absorption and blood–brain barrier permeation of compound **5f**. As shown in Figure 6**B**, compound **5f** was positioned outside the regions associated with high passive gastrointestinal absorption and BBB permeation, consistent with the **low GI absorption** and absence of predicted **BBB permeation** reported in Table 3. In addition, compound **5f** was predicted to be a **P-glycoprotein substrate**. This predicted efflux liability, together with its high lipophilicity and poor aqueous solubility, highlights potential limitations in its pharmacokinetic profile that should be considered during further structural optimization.

The potential biological target profile of compound **5f** was further explored using **SwissTargetPrediction**. As illustrated in Figure 6**C**, the predicted targets were distributed across several protein classes, including kinase-related targets. Notably, **EGFR was identified among the top 15 predicted targets for compound 5f**, providing additional computational support for examining its interaction with EGFR in the docking analysis. SwissTargetPrediction analysis was also performed for compounds **5a**–**5e** and **5g**–**5i**, and the corresponding target-class distributions are provided in the **Supplementary Material**. Across the investigated series, EGFR was recurrently identified among the predicted targets, although its relative ranking varied among individual derivatives. These predictions should be interpreted as exploratory computational estimates rather than evidence of experimentally confirmed target engagement.

Taken together, the Bioavailability Radar, BOILED-Egg, and SwissTargetPrediction analyses complement the molecular docking and pharmacokinetic prediction results by providing additional information on the physicochemical characteristics and potential target profile of compound **5f**. Although **5f** exhibited the most favorable docking score within the investigated series, its high predicted lipophilicity, poor aqueous solubility, low gastrointestinal absorption, P-glycoprotein substrate status, and two Lipinski rule violations indicate relevant drug-likeness and pharmacokinetic limitations. Therefore, compound **5f** represents a computationally prioritized derivative for further investigation rather than an optimized drug-like candidate. Its predicted interaction with EGFR should likewise be considered supportive in silico evidence, and **experimental biochemical and cellular studies are required to establish EGFR binding and inhibitory activity**.

### 3.4. Molecular Dynamics Simulation Analysis

To further investigate the dynamic behavior of the computationally prioritized protein–ligand complex, **three independent 100 ns molecular dynamics (MD) simulations** were performed for compound **5f in complex with EGFR (PDB ID: 1M17)**, providing a cumulative simulation time of **300 ns**. Compound **5f** was selected for MD analysis based on its most favorable docking score within the investigated series. Independent trajectories were generated to assess the reproducibility of the observed structural behavior and to avoid drawing conclusions from a single simulation.

The trajectories were analyzed using several complementary structural and interaction descriptors, including protein backbone root-mean-square deviation (**RMSD**), ligand RMSD after backbone fitting, Cα root-mean-square fluctuation (**RMSF**), radius of gyration (**Rg**), protein–ligand minimum distance, intermolecular contacts, and protein–ligand hydrogen bonds. These parameters were evaluated collectively to characterize protein structural behavior, ligand reorientation, and the persistence of protein–ligand association across the three independent **100 ns trajectories**. A comparative statistical summary of the calculated MD parameters for Replicates 1–3 is presented in **Table 4**.

The backbone RMSD profiles of the EGFR–5f complex obtained from three independent 100 ns MD simulations are shown in Figure 7. Replicate 1 exhibited an average RMSD of 0.282 ± 0.048 nm over the entire trajectory. During approximately the first 75–80 ns, the RMSD fluctuated predominantly around 0.24–0.30 nm, followed by a transition toward a higher-RMSD conformational state during the final part of the simulation, with an average value of 0.349 ± 0.035 nm over 80–100 ns. Replicate 2 showed a comparatively consistent profile throughout the simulation, with an overall RMSD of 0.280 ± 0.028 nm and reduced fluctuations during the final 20 ns (0.297 ± 0.013 nm). Replicate 3 displayed the lowest overall backbone deviation, with an average RMSD of 0.217 ± 0.030 nm, while the final 20 ns remained highly consistent at 0.210 ± 0.015 nm. Overall, the three independent simulations revealed replicate-dependent conformational behavior rather than identical trajectories. The comparatively limited backbone deviations observed across the simulations indicate that the overall EGFR backbone conformation was maintained over the simulated timescale, although Replicate 1 sampled an alternative higher-RMSD conformational state toward the end of the trajectory.

Residue-level flexibility of the EGFR–5f complex was evaluated by Cα root-mean-square fluctuation (RMSF) analysis across the three independent 100 ns MD trajectories (Figure 8). The mean RMSF values were 0.1367 ± 0.1658 nm, 0.1410 ± 0.1256 nm, and 0.1082 ± 0.0872 nm for Replicates 1, 2, and 3, respectively. The corresponding overall RMSF ranges were 0.0451–1.2422 nm, 0.0479–1.0682 nm, and 0.0399–0.8087 nm. The largest fluctuations were concentrated predominantly at the terminal regions of the protein, with maximum values of 1.2422, 1.0682, and 0.8087 nm in Replicates 1–3, respectively. In contrast, most residues within the structured protein core exhibited substantially smaller fluctuations, generally remaining within approximately 0.05–0.15 nm, with several localized regions showing moderate increases. The overall shapes of the RMSF profiles were broadly reproduced across the independent trajectories, although Replicate 2 displayed somewhat greater local flexibility in selected regions, whereas Replicate 3 generally exhibited lower fluctuation amplitudes. These results indicate that the elevated RMSF values were primarily associated with flexible terminal regions rather than widespread increases in backbone mobility, while the structured protein core remained comparatively less flexible throughout the three 100 ns trajectories.

The radius of gyration (Rg) was evaluated to assess the global compactness of the EGFR–5f complex during the three independent 100 ns MD simulations (Figure 9). The mean Rg values were 2.0201 ± 0.0156 nm, 2.0396 ± 0.0157 nm, and 2.0047 ± 0.0122 nm for Replicates 1, 2, and 3, respectively. During the final 20 ns, the corresponding mean values were 2.0338 ± 0.0100 nm, 2.0275 ± 0.0112 nm, and 2.0026 ± 0.0077 nm. The overall Rg ranges were 1.9802–2.0729 nm, 1.9983–2.1055 nm, and 1.9686–2.0612 nm, respectively. Although Replicate 2 displayed slightly higher Rg values during parts of the trajectory, no progressive increase in Rg was observed in any of the three simulations. These results indicate that the overall compactness of the EGFR structure was maintained over the simulated timescale, with only modest replicate-dependent conformational fluctuations.

Protein–ligand hydrogen bonding was evaluated throughout the three independent 100 ns MD simulations to characterize the persistence of polar interactions between **5f** and EGFR (Figure 10). The average numbers of protein–ligand hydrogen bonds were 0.8705 ± 0.3411, 2.1082 ± 0.6971, and 1.0307 ± 0.4209 for Replicates 1, 2, and 3, respectively. During the final 20 ns, the corresponding values were 0.9875 ± 0.1239, 2.4563 ± 0.6559, and 0.9980 ± 0.0948. At least one protein–ligand hydrogen bond was present in approximately 86.9%, 96.7%, and 94.4% of the analyzed frames in Replicates 1–3, respectively. Replicates 1 and 3 predominantly sampled configurations containing approximately one hydrogen bond, whereas Replicate 2 more frequently exhibited two to three simultaneous hydrogen bonds. These results demonstrate sustained, but replicate-dependent, hydrogen-bonding interactions between **5f** and the EGFR binding site over the three 100 ns simulation trajectories.

The minimum protein–ligand distance was monitored throughout the three independent 100 ns MD simulations to assess the persistence of close intermolecular contacts between **5f** and EGFR (Figure 11). The average minimum distances were 0.1914 ± 0.0136 nm, 0.1779 ± 0.0121 nm, and 0.1908 ± 0.0146 nm for Replicates 1–3, respectively. During the final 20 ns, the corresponding mean values remained at 0.1872 ± 0.0124 nm, 0.1771 ± 0.0103 nm, and 0.1897 ± 0.0124 nm. No progressive increase in the minimum protein–ligand distance was observed in any of the three trajectories, indicating that **5f** remained in close contact with EGFR throughout the simulated timescale.

The number of protein–ligand atomic contacts within 0.35 nm was also evaluated (Figure 12). The mean numbers of close contacts were 141.62 ± 20.49, 129.26 ± 26.82, and 137.03 ± 24.99 for Replicates 1–3, respectively. During the final 20 ns, the corresponding values were 148.44 ± 17.91, 110.14 ± 18.53, and 154.45 ± 20.41. Replicates 1 and 3 retained or increased the number of close contacts during the final portion of the trajectories, whereas Replicate 2 showed a reduction in total atomic contacts. Importantly, this decrease was not accompanied by an increase in the minimum protein–ligand distance and coincided with persistent hydrogen bonding, suggesting a rearrangement of the ligand–binding-site interaction pattern rather than ligand dissociation. Overall, the contact analysis supports sustained association of **5f** with EGFR while also indicating replicate-dependent binding-site reorganization.

Ligand RMSD was calculated after least-squares fitting of each trajectory to the protein backbone in order to evaluate changes in the position and conformation of **5f** relative to its initial binding pose (Figure 13). The mean ligand RMSD values were 0.4146 ± 0.0942 nm, 0.8060 ± 0.0973 nm, and 0.8623 ± 0.3232 nm for Replicates 1, 2, and 3, respectively. During the final 20 ns, the corresponding values were 0.3919 ± 0.0530 nm, 0.8389 ± 0.0607 nm, and 0.9930 ± 0.0685 nm. Replicate 1 remained comparatively close to the initial ligand pose, whereas Replicates 2 and 3 sampled more displaced binding configurations. In particular, Replicate 3 displayed several transitions between distinct RMSD levels before reaching a comparatively consistent plateau during the final portion of the trajectory. Importantly, the larger ligand RMSD values observed in Replicates 2 and 3 were not accompanied by increased protein–ligand separation. The minimum-distance, contact, and hydrogen-bond analyses confirmed continued association of **5f** with EGFR. Thus, the ligand RMSD behavior is more consistent with replicate-dependent reorientation and binding-site rearrangement than with ligand dissociation.

Taken together, the three independent 100 ns simulations revealed reproducible preservation of the overall EGFR backbone architecture, while also demonstrating replicate-dependent conformational sampling and ligand reorientation. Despite differences in ligand RMSD and contact patterns among the trajectories, the consistently short protein–ligand minimum distances, sustained hydrogen-bonding interactions, and maintenance of close intermolecular contacts indicated continued association of **5f** with the EGFR binding site throughout the simulated timescale. These findings therefore support structural persistence of the complex over the investigated trajectories while avoiding interpretation of the MD results as evidence of definitive thermodynamic stability.

## 4. Conclusions

The present study provides an integrated computational characterization of a series of previously synthesized carbazole-based urea and thiourea derivatives (**5a**–**5i**), with particular emphasis on their predicted interaction with **EGFR tyrosine kinase** and the identification of physicochemical factors that may influence their further development. Rather than establishing a uniform substituent-dependent trend across the series, the docking results revealed a compound-specific response to structural modification. Among the investigated derivatives, **5f** was prioritized on the basis of its favorable predicted interaction with the EGFR binding site. Redocking of the co-crystallized ligand supported the ability of the applied docking protocol to reproduce the experimentally observed binding orientation, strengthening the basis for comparative interpretation of the docking results.

Detailed examination of **5f** indicated that its predicted interaction with EGFR involves a combination of hydrogen-bonding, hydrophobic, π-related, halogen, and van der Waals contacts. Importantly, the complementary **SwissTargetPrediction** analysis also identified EGFR within the predicted target profile of 5f. Although this convergence between independent computational approaches strengthens the rationale for considering EGFR as a relevant target for further investigation, it does not establish direct target engagement or inhibitory activity and therefore requires experimental validation.

The physicochemical and pharmacokinetic predictions revealed a clear limitation of the series. The favorable docking behavior of **5f** was accompanied by high lipophilicity, poor predicted aqueous solubility, low gastrointestinal absorption, P-glycoprotein substrate behavior, and multiple Lipinski rule violations. Thus, improved predicted receptor interaction did not translate into an optimal drug-likeness profile. This balance between target-oriented molecular recognition and unfavorable physicochemical properties represents an important consideration for subsequent optimization of this scaffold. Future structural modifications should therefore aim not simply to preserve favorable EGFR interactions but also to reduce excessive lipophilicity and improve solubility and predicted absorption.

The **three independent 100 ns molecular dynamics simulations** provided an extended dynamic assessment of the EGFR–5f complex. Across the trajectories, the overall EGFR backbone conformation and compactness were maintained over the simulated timescale, while **5f** exhibited replicate-dependent reorientation within the binding site. Despite differences in ligand RMSD and contact patterns among the replicates, consistently short protein–ligand minimum distances, sustained hydrogen-bonding interactions, and the persistence of close intermolecular contacts indicated continued association of **5f** with EGFR throughout the simulations. These observations therefore support **structural persistence and continued protein–ligand association over the investigated 100 ns trajectories**, while not constituting evidence of definitive thermodynamic stability.

Taken together, the results position compound **5f as a computationally prioritized member of the series rather than a validated EGFR inhibitor or an optimized drug candidate**. The principal value of the present analysis lies in defining both the favorable molecular-recognition features and the physicochemical liabilities that should guide subsequent development. Experimental EGFR binding or kinase-inhibition assays, followed by cellular evaluation, will be essential to determine whether the predicted interaction profile translates into measurable biological activity. If such activity is confirmed, optimization of the scaffold should focus on improving solubility and pharmacokinetic properties while retaining the interaction features identified in the present computational analysis.

## Figures and Tables

**Figure 1:**
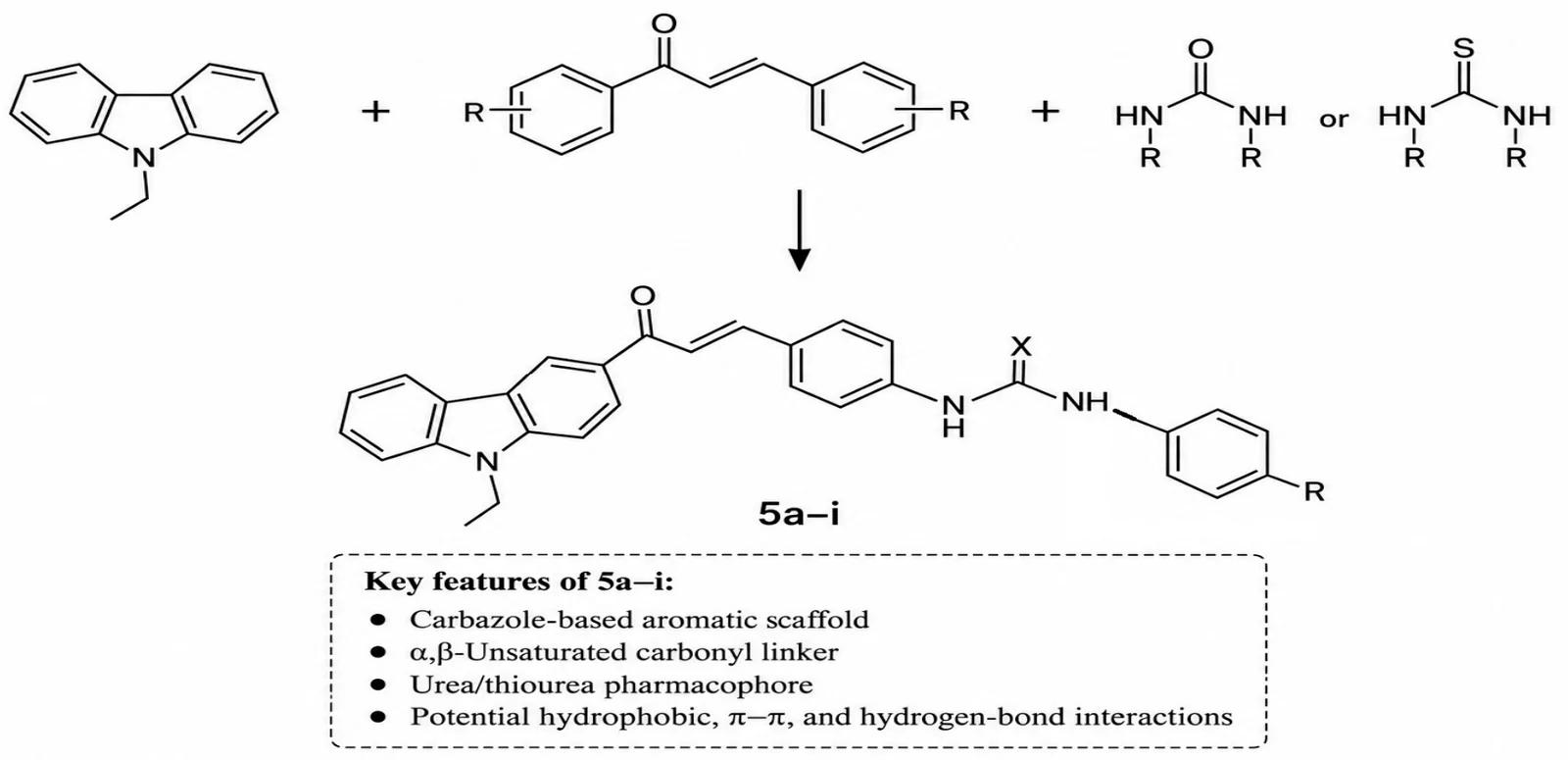
Schematic representation of the molecular hybridization strategy used for the design of carbazole–chalcone urea/thiourea hybrids **5a**–**i**.

**Scheme 1:**
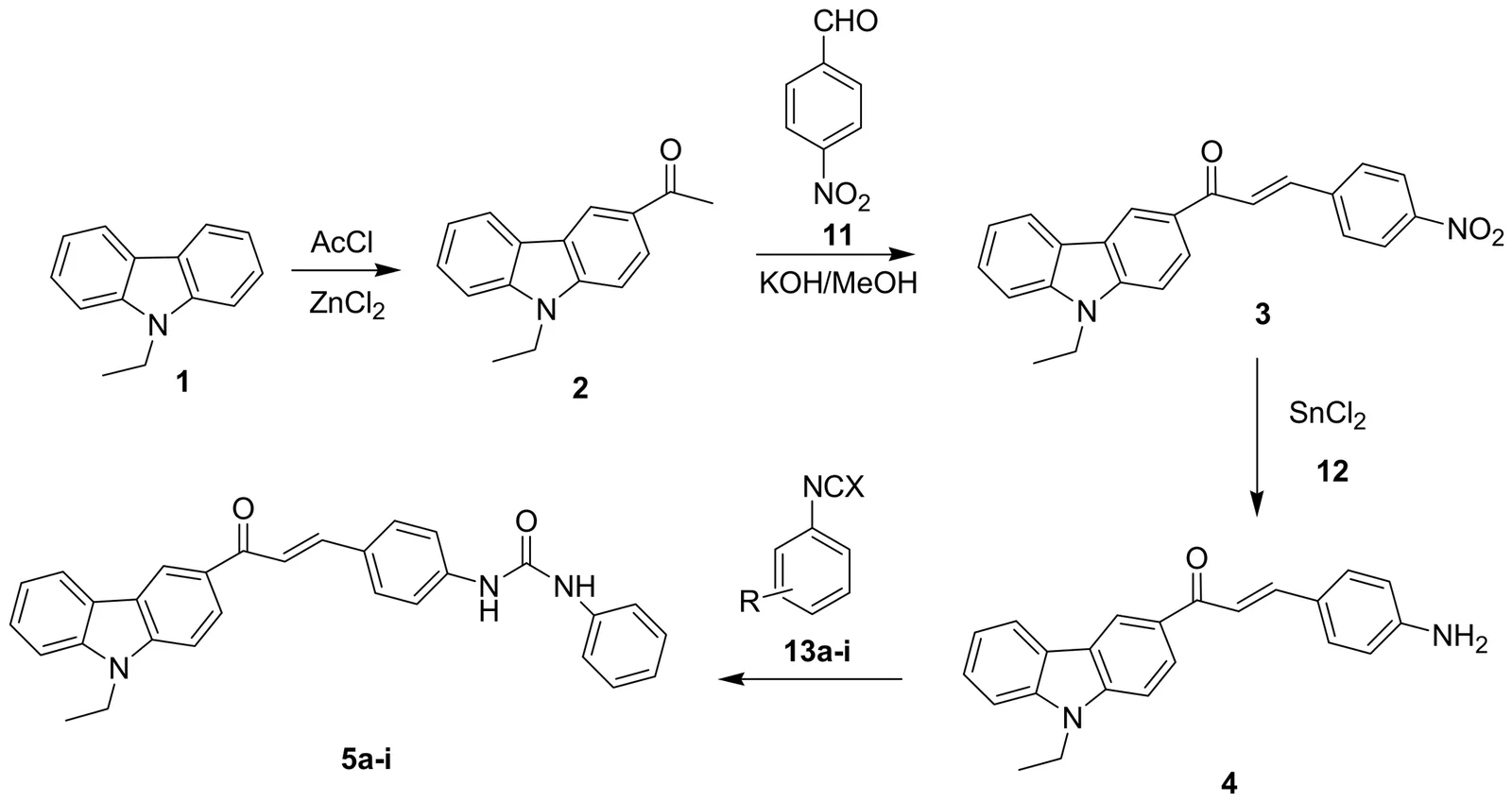
Synthetic route to compounds **5a**–**5i** based on the previously reported procedure [48]. Reagents and conditions: (i) AcCl, ZnCl_2_; (ii) 4-nitrobenzaldehyde, MeOH, 20% aqueous KOH, room temperature, 24 h; (iii) SnCl_2_, THF, reflux, 4 h; (iv) corresponding aryl isocyanate or isothiocyanate derivative, dry DMF, 40 °C, 6 h.

**Figure 2:**
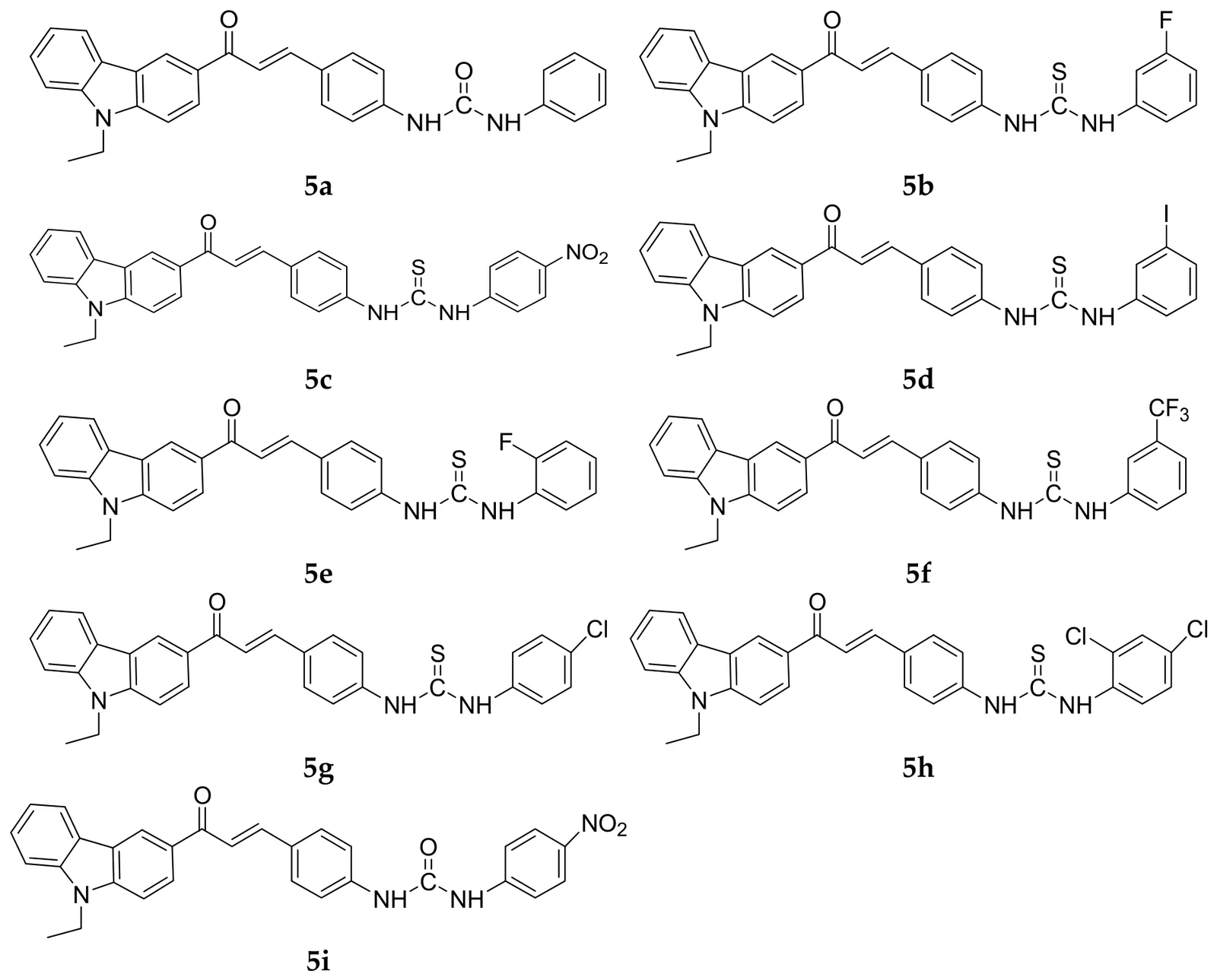
Chemical structures of compounds **5a**–**5i** investigated in the present computational study.

**Figure 3:**
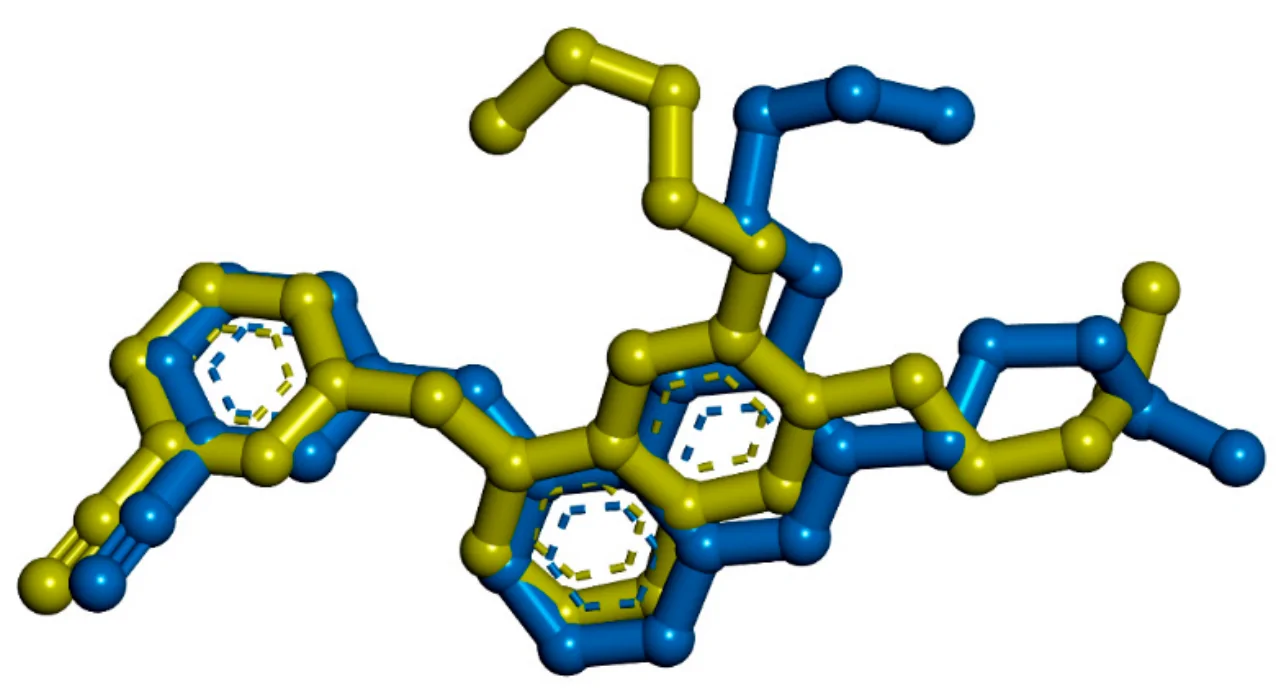
Validation of the molecular docking protocol by re-docking the co-crystallized ligand AQ4 into the EGFR binding site (PDB ID: 1M17). Superposition of the crystallographic AQ4 pose (yellow) and the selected re-docked pose, Mode 3 (blue), yielded an RMSD of 1.65 Å and a docking score of −6.8 kcal/mol.

**Figure 4:**
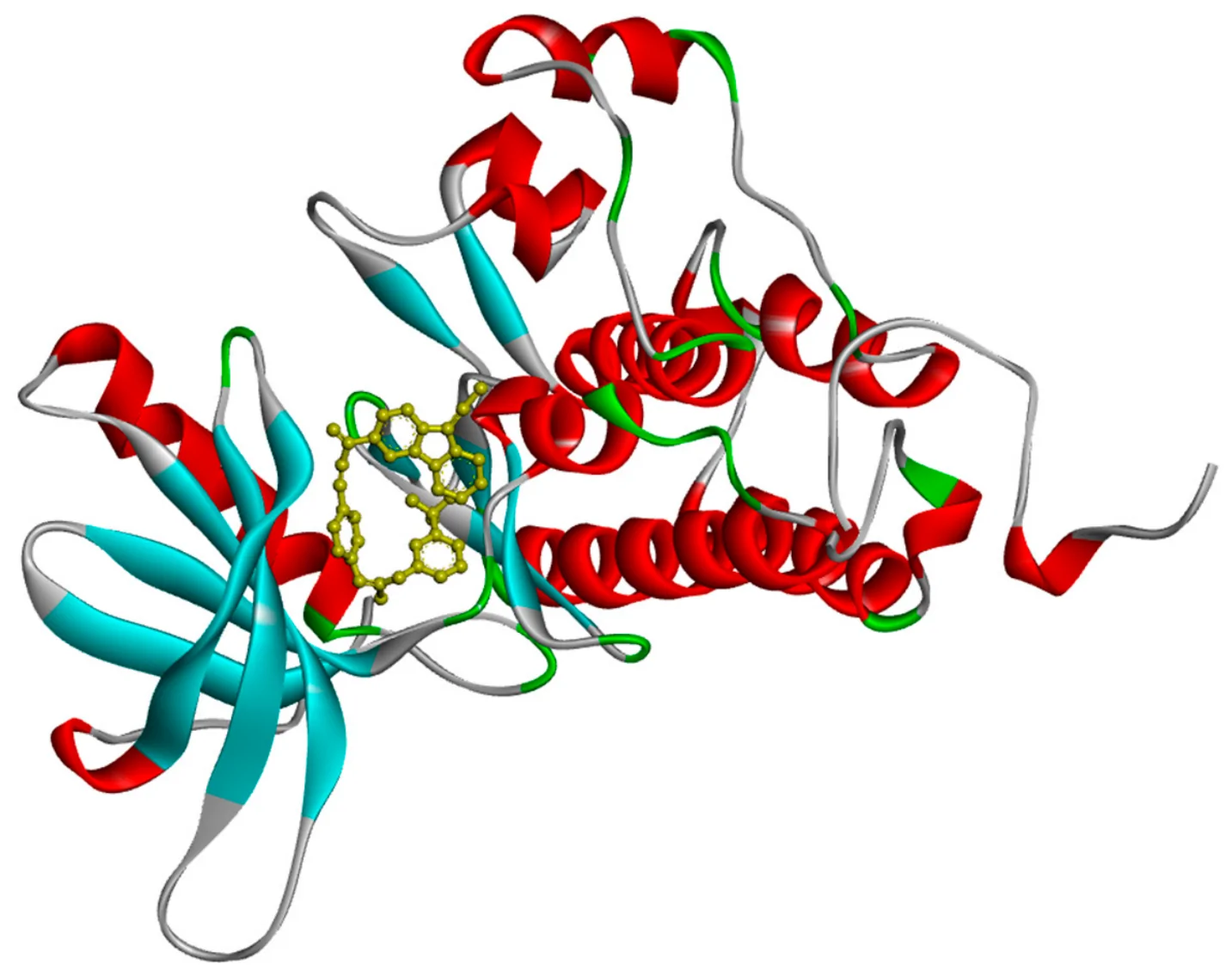
Three-dimensional representation of the predicted binding pose of compound **5f** within the active site of EGFR tyrosine kinase (PDB ID: 1M17). The protein is shown in cartoon representation, with α-helices in red, β-sheets in cyan, and loop/coil regions in green and gray; compound **5f** is shown in yellow.

**Figure 5:**
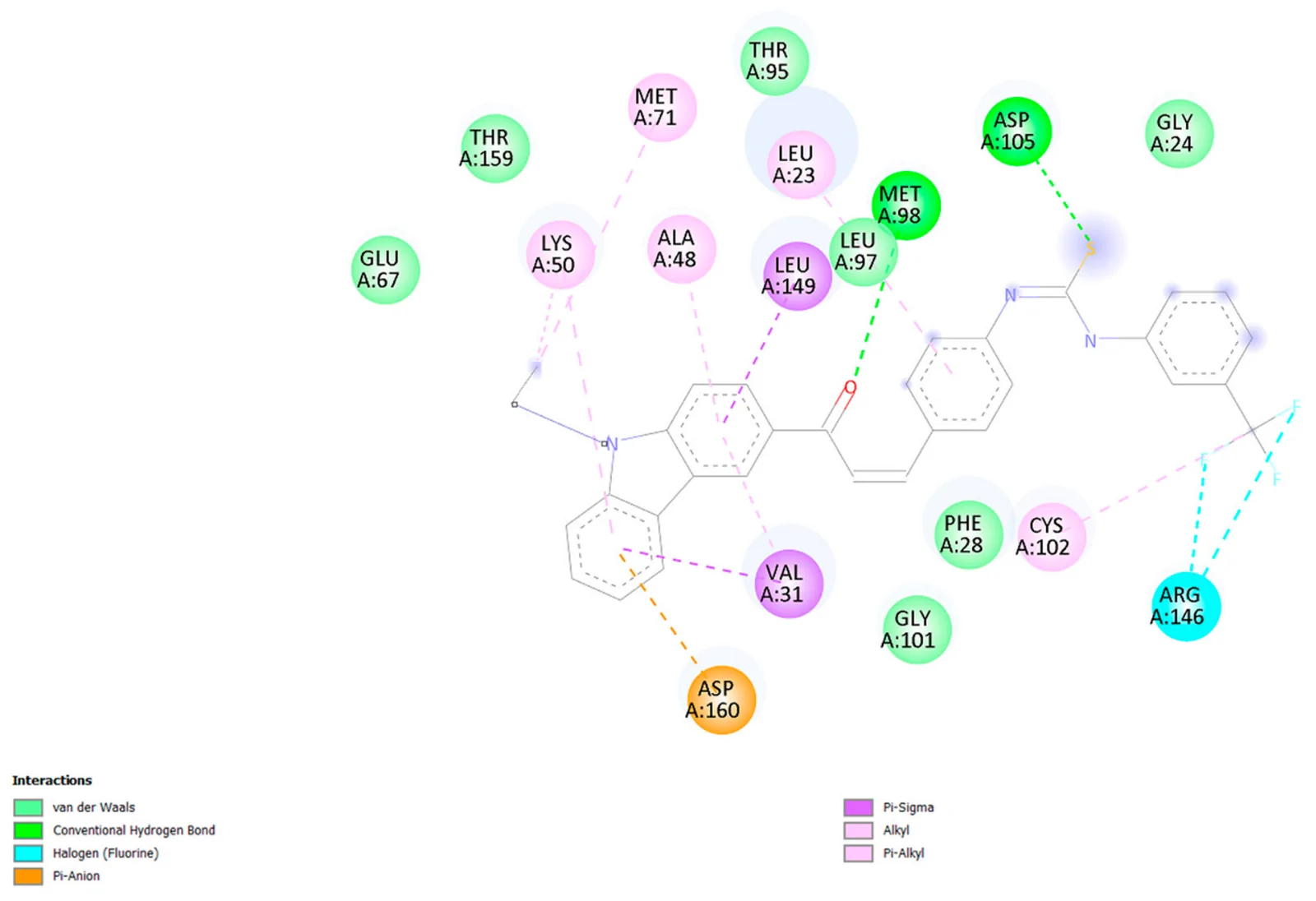
Two-dimensional interaction diagram of compound **5f** within the binding pocket of EGFR (PDB ID: 1M17), showing the predicted hydrogen-bonding, hydrophobic, π-related, halogen, and van der Waals interactions with surrounding amino acid residues.

**Figure 6:**
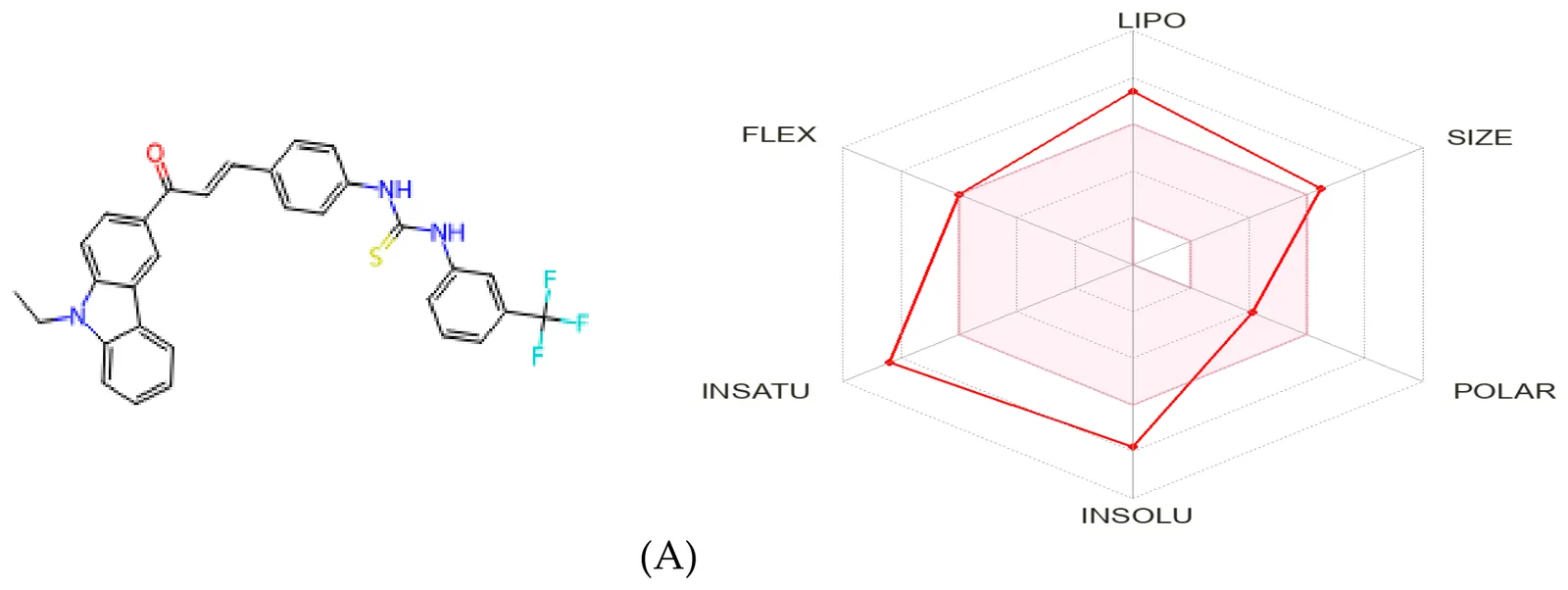

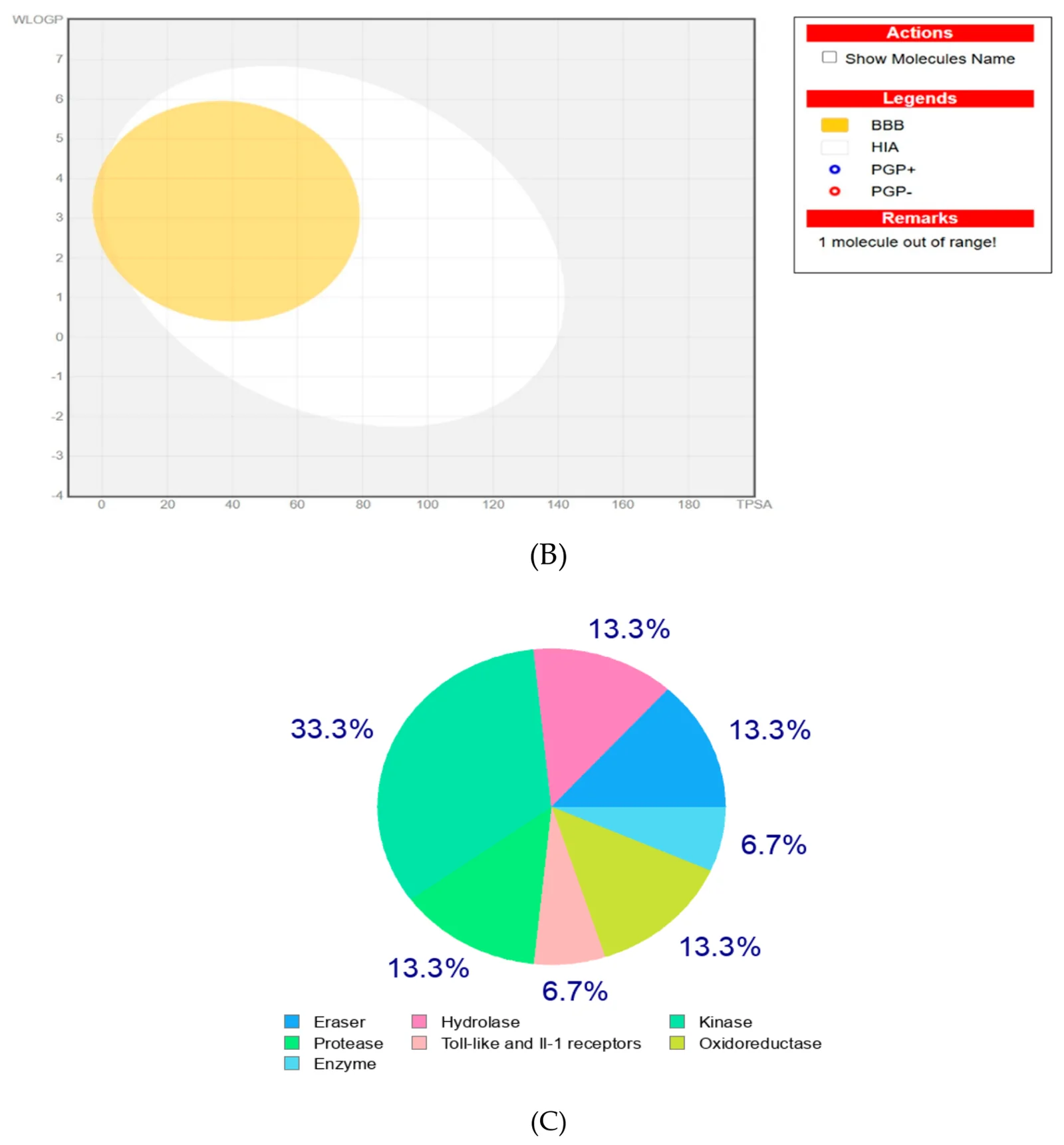
Computational prediction profiles of compound **5f**: (**A**) Bioavailability Radar illustrating six physicochemical properties relevant to oral drug-likeness: lipophilicity (LIPO), molecular size (SIZE), polarity (POLAR), solubility (INSOLU), flexibility (FLEX), and saturation (INSATU); (**B**) BOILED-Egg model illustrating the predicted passive gastrointestinal absorption, blood–brain barrier (BBB) permeation, and P-glycoprotein (P-gp) substrate status; and (**C**) SwissTargetPrediction target-class distribution based on the top 15 predicted biological targets.

**Figure 7:**
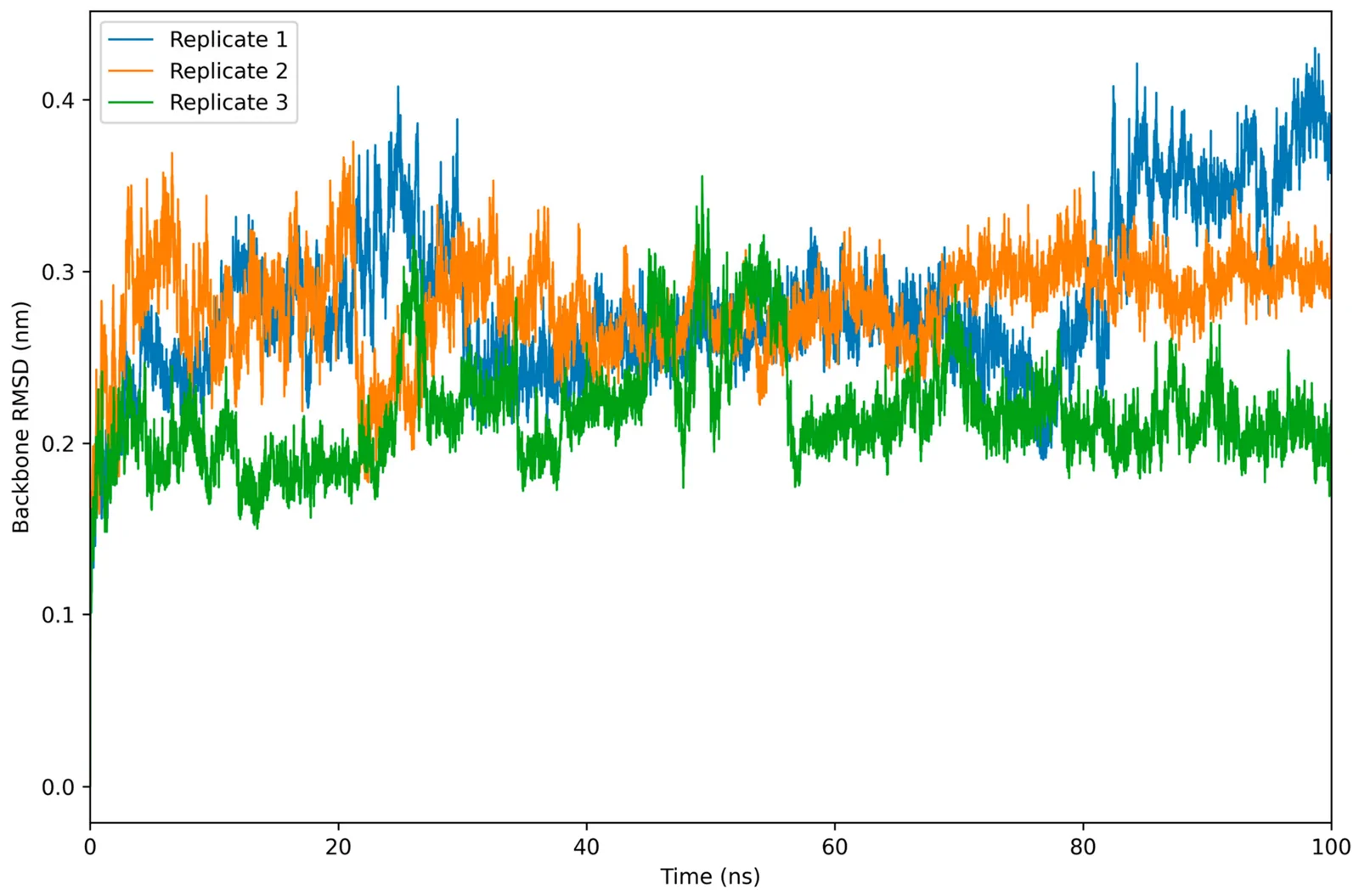
Backbone RMSD profiles of the EGFR–5f complex obtained from three independent 100 ns molecular dynamics simulations. RMSD values were calculated after least-squares fitting of each trajectory to the corresponding starting protein backbone.

**Figure 8:**
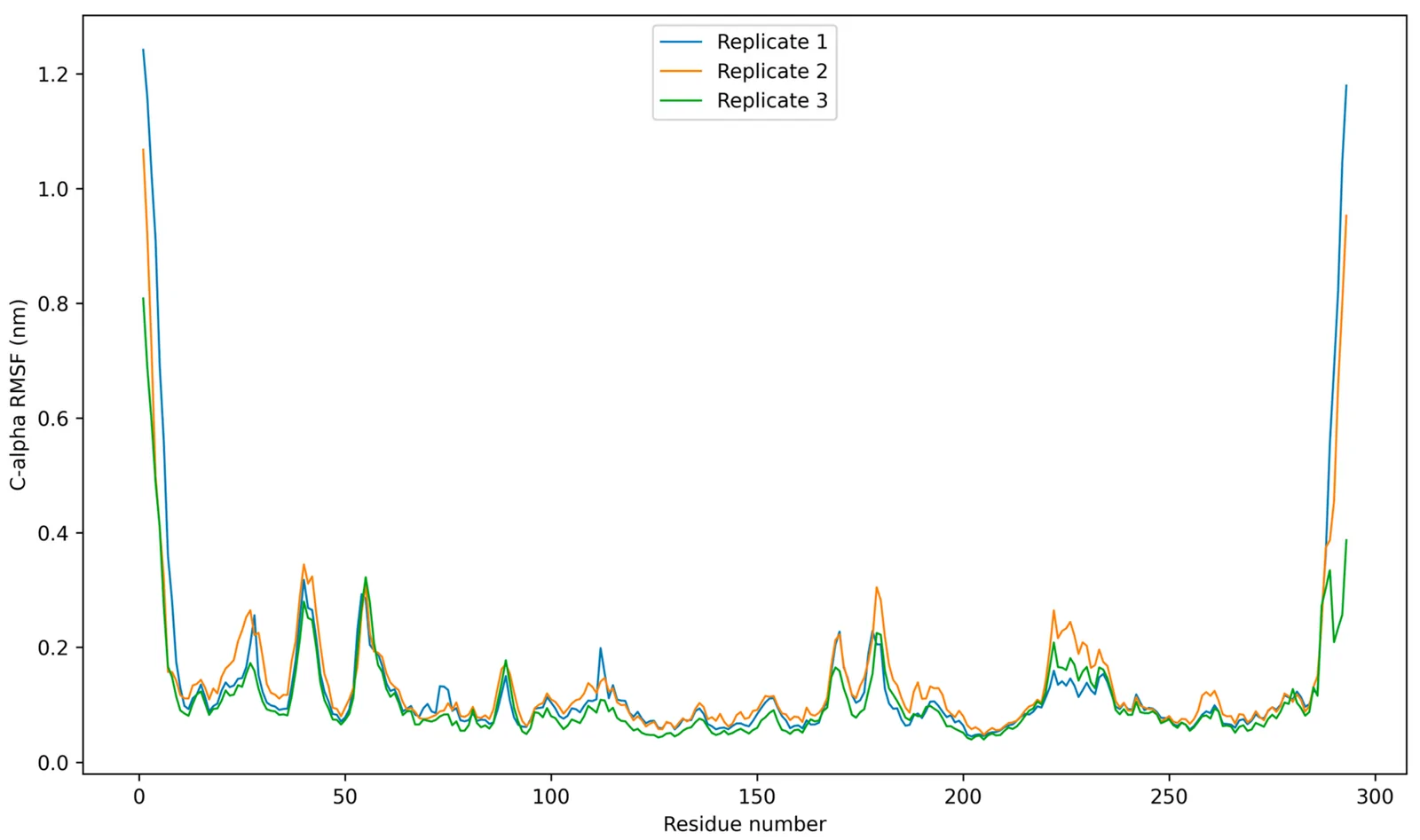
Cα RMSF profiles of the EGFR–5f complex obtained from three independent 100 ns molecular dynamics simulations. RMSF values represent residue-wise fluctuations calculated over each trajectory.

**Figure 9:**
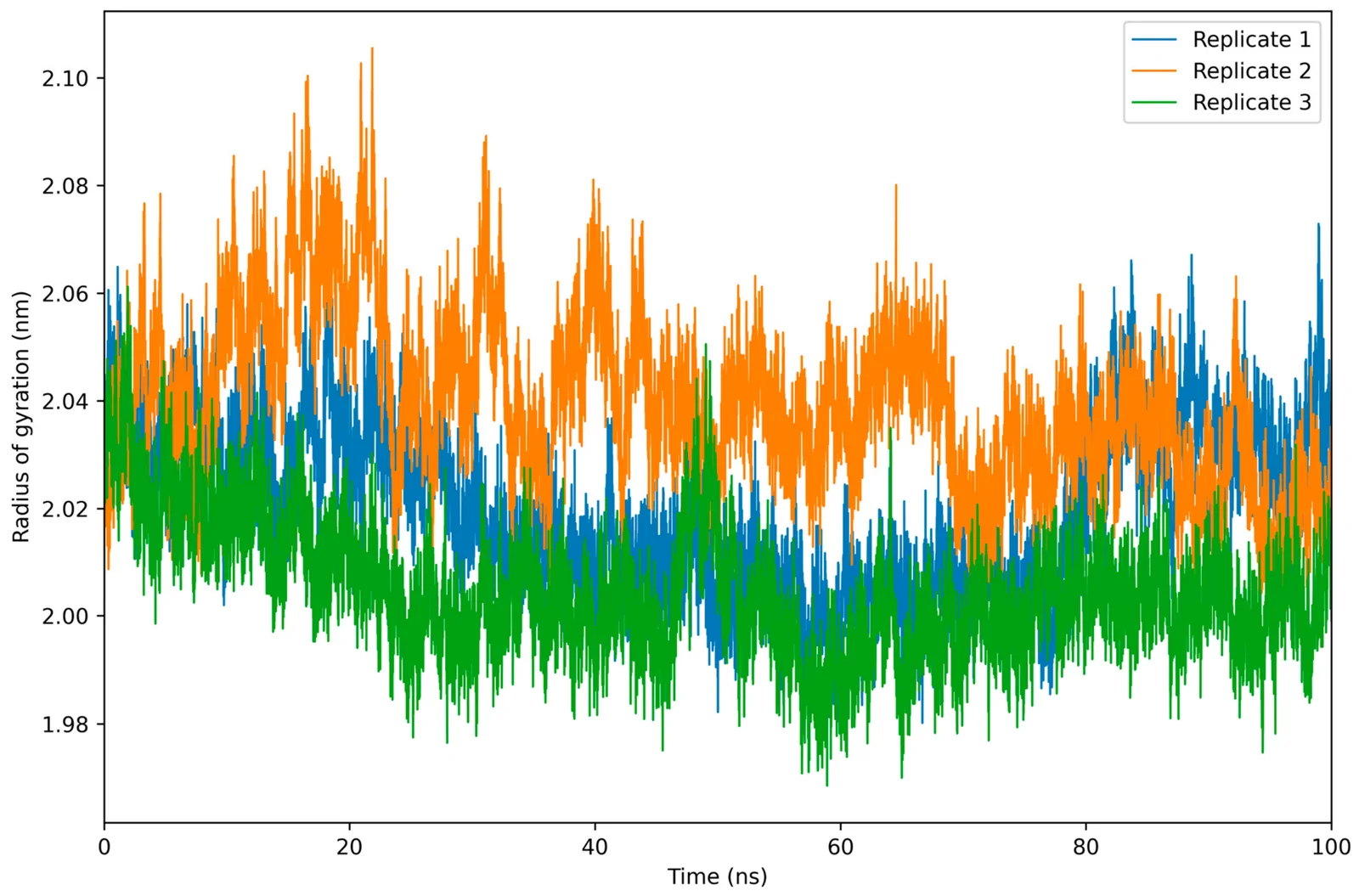
Radius of gyration (Rg) profiles of the EGFR–5f complex obtained from three independent 100 ns molecular dynamics simulations.

**Figure 10:**
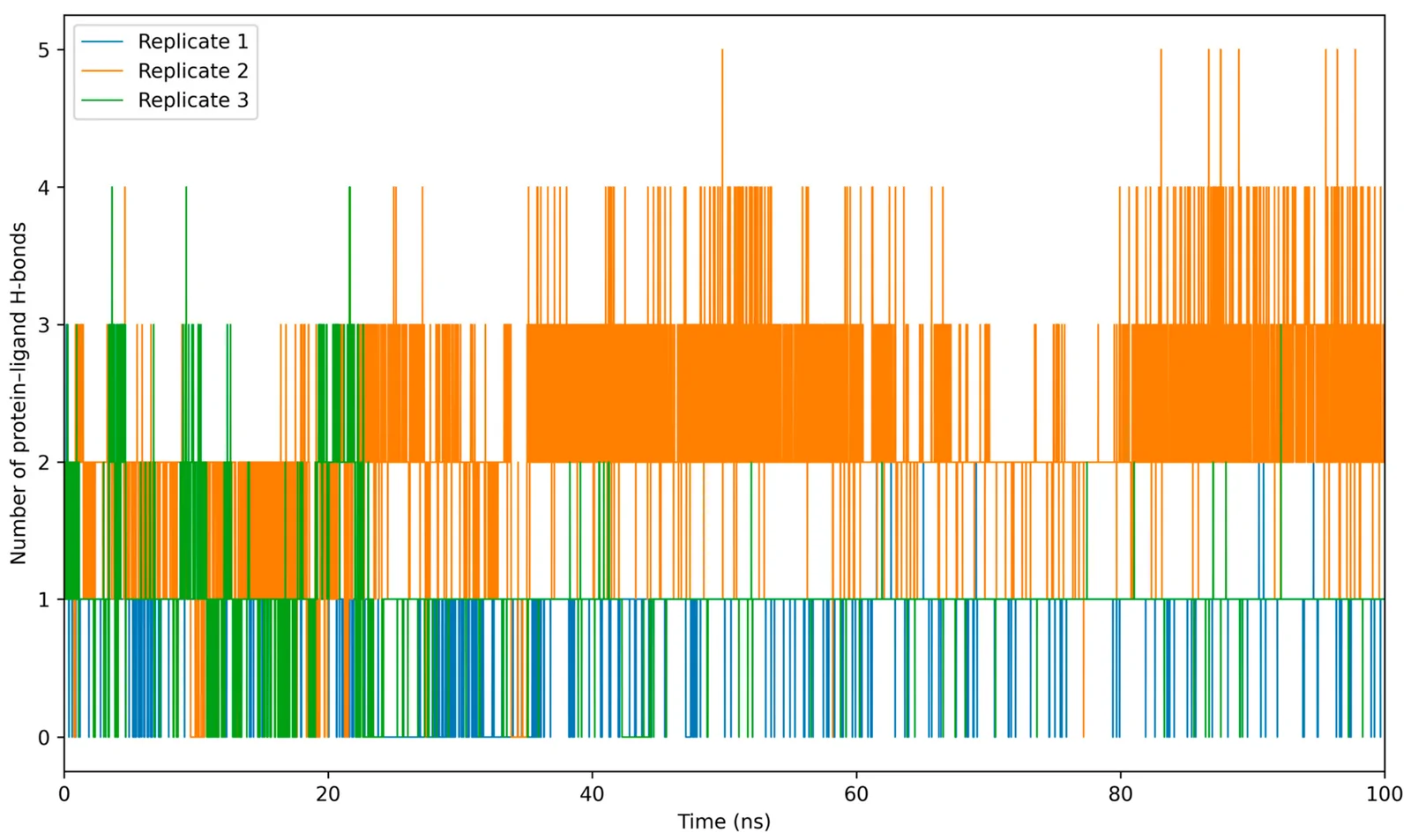
Protein–ligand hydrogen-bond profiles of the EGFR–5f complex obtained from three independent 100 ns molecular dynamics simulations.

**Figure 11:**
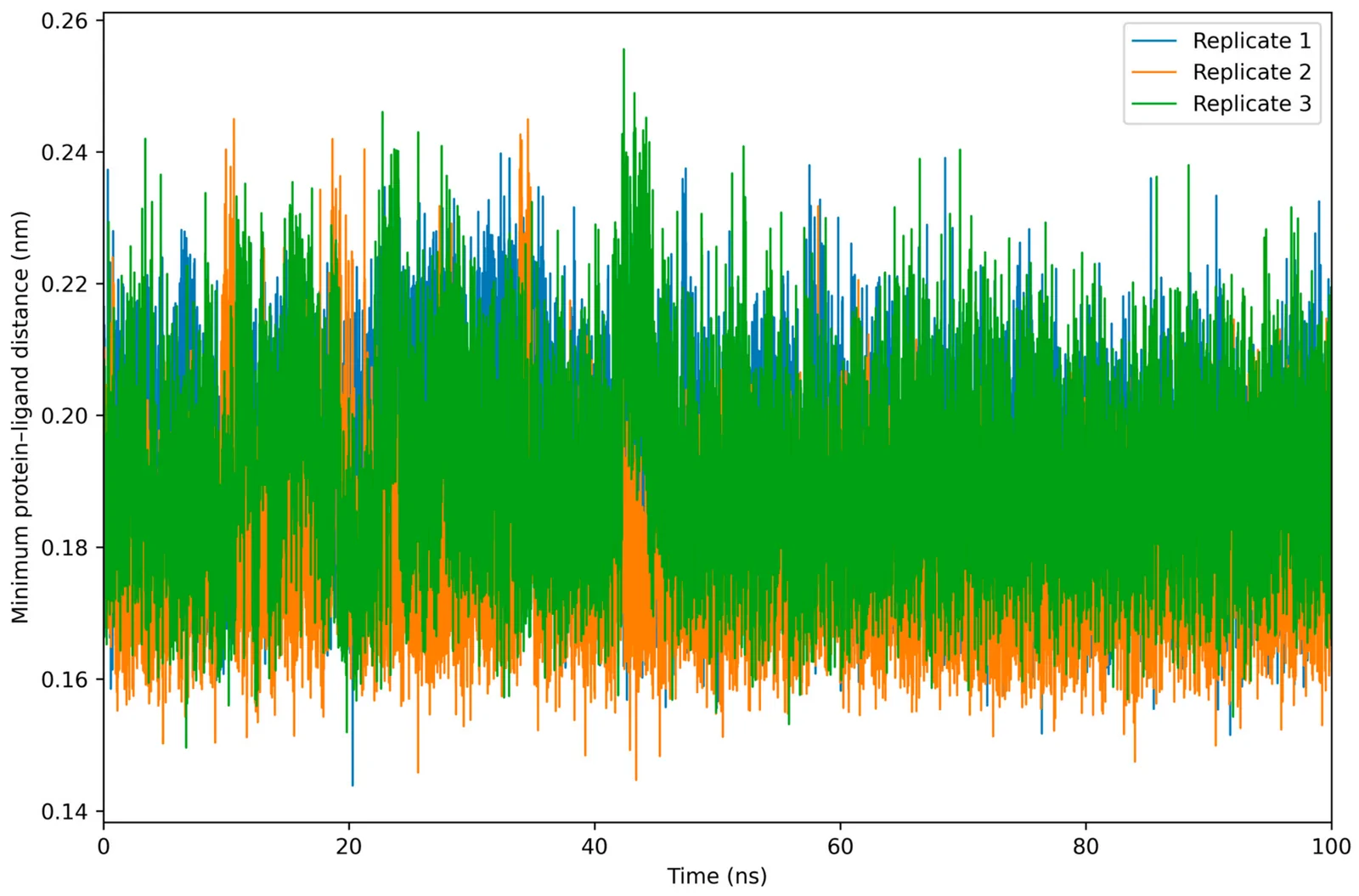
Minimum protein–ligand distance profiles for the EGFR–5f complex obtained from three independent 100 ns molecular dynamics simulations.

**Figure 12:**
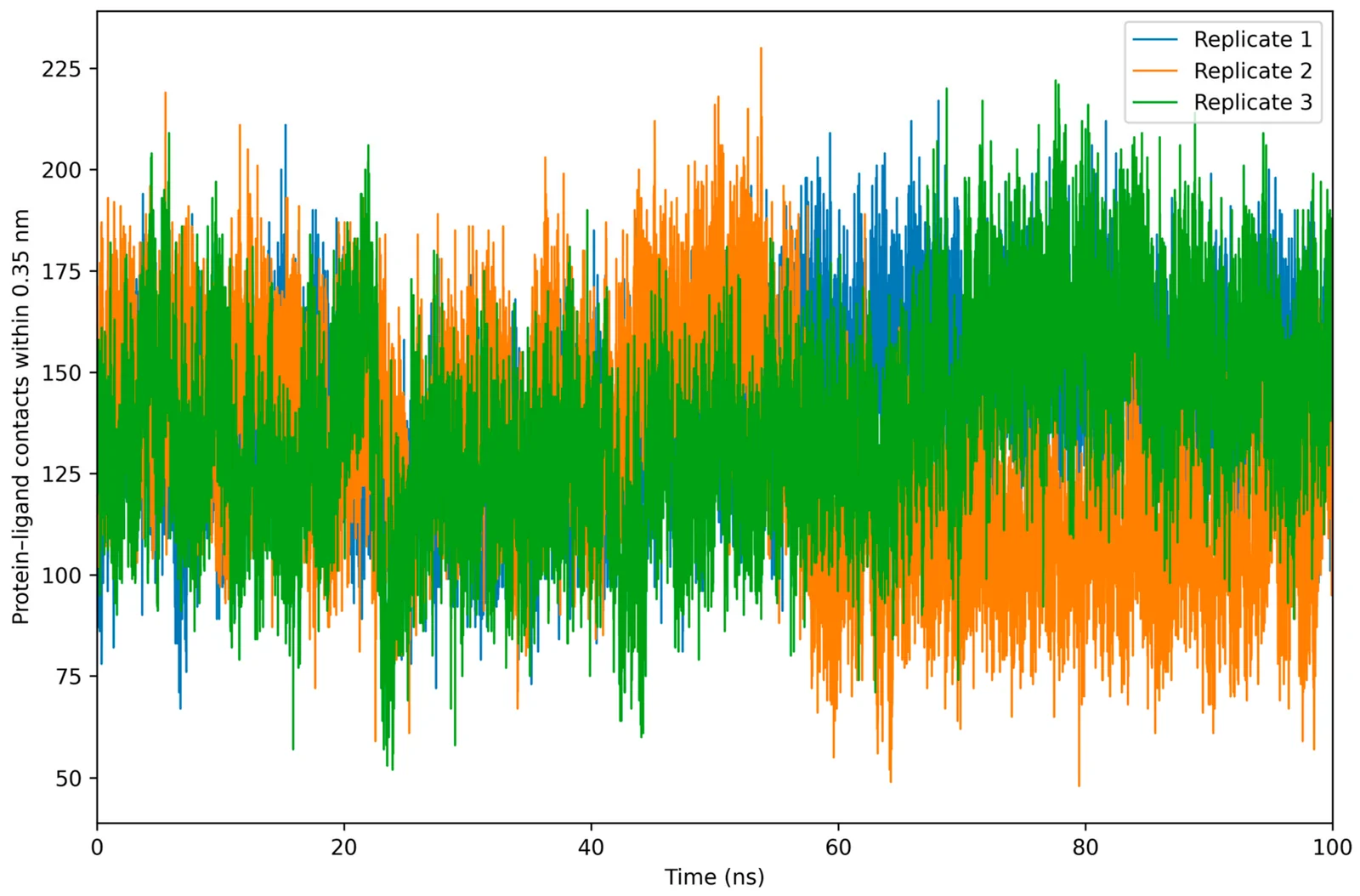
Protein–ligand atomic contact profiles for the EGFR–5f complex obtained from three independent 100 ns molecular dynamics simulations. Contacts were defined using a distance cutoff of 0.35 nm.

**Figure 13:**
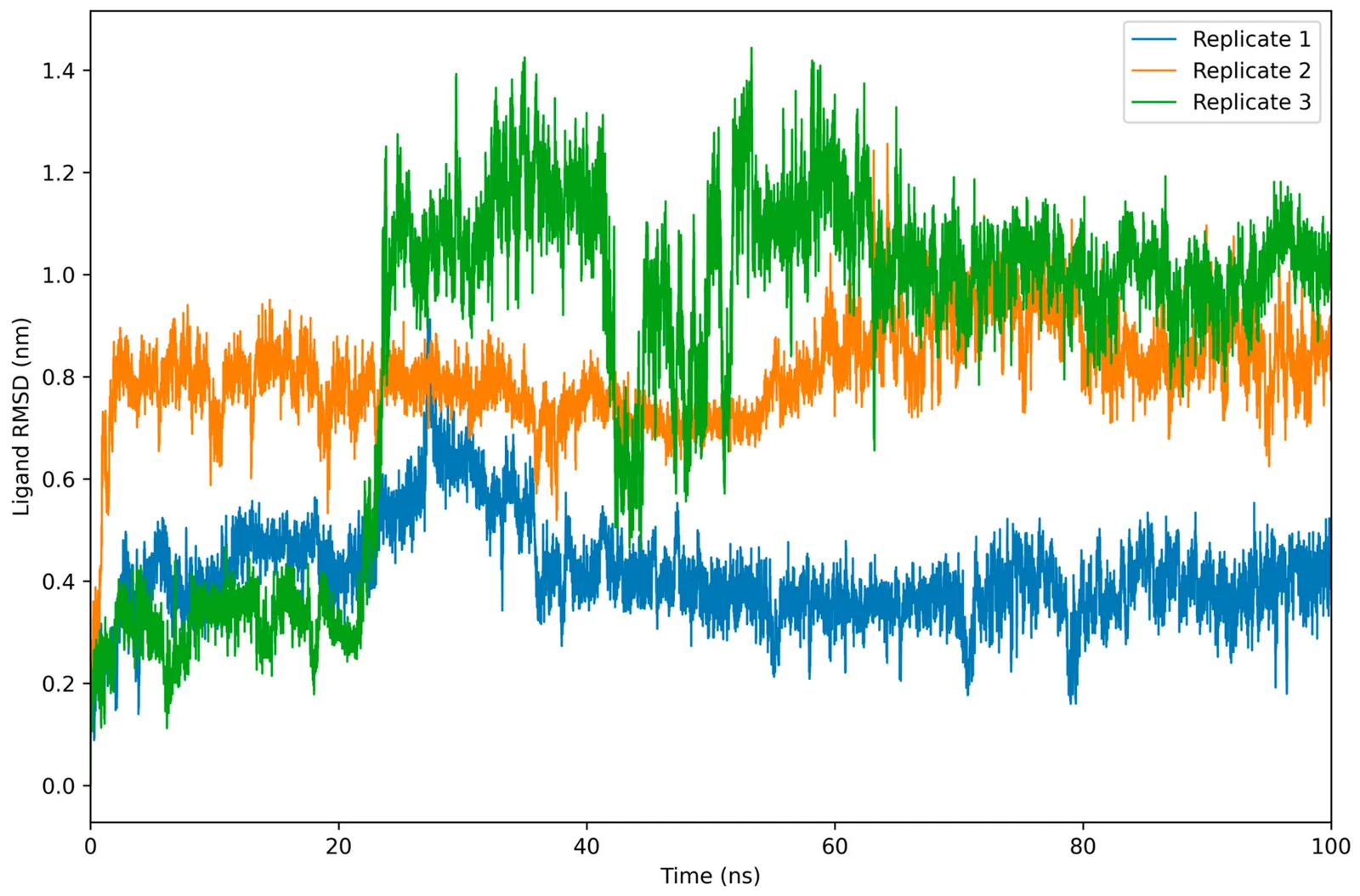
Ligand RMSD profiles of 5f in complex with EGFR obtained from three independent 100 ns molecular dynamics simulations. Ligand RMSD values were calculated after least-squares fitting of each trajectory to the protein backbone.

**Table 1:** R and X assignments for compounds **5a**–**5i**. X = O denotes urea derivatives, whereas X = S denotes thiourea derivatives.

Compound ID	R	X
**5a**	H	O
**5b**	3-F	S
**5c**	4–NO_2_	S
**5d**	3-I	S
**5e**	2-F	S
**5f**	3–CF_3_	S
**5g**	4-Cl	S
**5h**	2,4–Cl_2_	S
**5i**	4–NO_2_	O

**Table 2:** Molecular docking scores of compounds **5a**–**5i** and the selected AQ4 re-docking validation pose against EGFR tyrosine kinase (PDB ID: 1M17).

Compound	Substituent	Docking Score (kcal/mol)
**5f**	3-CF_3_	−13.5
**5c**	4-NO_2_ (thiourea)	−13.1
**5i**	4-NO_2_ (urea)	−13.1
**5e**	2-F	−12.9
**5g**	4-Cl	−12.8
**5h**	2,4-Cl_2_	−12.8
**5d**	3-I	−11.6
**5a**	H	−10.0
**5b**	3-F	−9.0
AQ4 (co-crystallized ligand)	Reference ligand	−6.8

**Table 3:** Predicted ADMET and drug-likeness properties of compounds **5a**–**5i**.

Comp.	MW (g/mol)	Consensus LogP	TPSA (Å^2^)	HBA	HBD	RB	GI Abs.	BBB	P-gp	Lipinski Viol.	Bioavail. Score	ESOL LogS	PAINS	Brenk	SA
**5a**	459.54	5.38	63.13	2	2	8	Low	No	No	1	0.55	−6.45	0	1	3.36
**5b**	493.59	6.19	78.15	2	2	8	Low	No	No	1	0.55	−7.09	0	2	3.49
**5c**	520.60	4.99	123.97	5	2	9	Low	No	No	1	0.55	−6.85	0	2	3.58
**5d**	601.50	6.55	78.15	2	2	8	Low	No	No	2	0.17	−8.81	0	2	3.68
**5e**	493.59	6.19	78.15	2	2	8	Low	No	No	1	0.55	−7.09	0	2	3.51
**5f**	543.60	7.05	78.15	2	2	9	Low	No	Yes	2	0.17	−7.78	0	2	3.65
**5g**	510.05	6.43	78.15	2	2	8	Low	No	No	2	0.17	−7.52	0	2	3.45
**5h**	544.49	6.92	78.15	2	2	8	Low	No	No	2	0.17	−8.12	0	2	3.61
**5i**	504.54	4.44	108.95	5	2	9	Low	No	No	1	0.55	−6.51	0	2	3.57

Abbreviations: MW, molecular weight; TPSA, topological polar surface area; HBAs, hydrogen bond acceptors; HBDs, hydrogen bond donors; RBs, rotatable bonds; GI Abs., gastrointestinal absorption; BBB, blood–brain barrier permeation; P-gp, P-glycoprotein substrate; ESOL LogS, estimated aqueous solubility; PAINS, pan-assay interference alerts; Brenk, Brenk structural alerts; SA, synthetic accessibility score.

**Table 4:** Summary of molecular dynamics parameters for the EGFR–5f complex obtained from three independent 100 ns MD simulations.

Parameter	Replicate 1	Replicate 2	Replicate 3
Protein backbone RMSD (nm)	0.282 ± 0.048	0.280 ± 0.028	0.217 ± 0.030
Ligand RMSD (nm)	0.4146 ± 0.0942	0.8060 ± 0.0973	0.8623 ± 0.3232
Protein Cα RMSF (nm)	0.1367 ± 0.1658	0.1410 ± 0.1256	0.1082 ± 0.0872
Minimum protein–ligand distance (nm)	0.1914 ± 0.0136	0.1779 ± 0.0121	0.1908 ± 0.0146
Contacts < 0.35 nm	141.62 ± 20.49	129.26 ± 26.82	137.03 ± 24.99
Protein–ligand H-bonds	0.8705 ± 0.3411	2.1082 ± 0.6971	1.0307 ± 0.4209
Radius of gyration (nm)	2.0201 ± 0.0156	2.0396 ± 0.0157	2.0047 ± 0.0122

Notes: Values are expressed as mean ± SD for each independent 100 ns MD replicate. Ligand RMSD was calculated after backbone fitting. Protein–ligand contacts were defined using a 0.35 nm distance cutoff.

## Data Availability

Data are contained within the article and Supplementary Materials.

## Supplementary Materials

The following supporting information can be downloaded at: https://www.mdpi.com/article/10.3390/molecules31193372/s1.
