# Supplementary material for: Computational Evaluation of Carbazole–Chalcone Urea and Thiourea Derivatives Against EGFR: Molecular Docking, Pharmacokinetic Profiling, Target Prediction, and Molecular Dynamics Simulation
Source: Molecules. 2026 Sep 23;31(19):3372. doi: 10.3390/molecules31193372 (PMC13666061; doi:10.3390/molecules31193372)
Supplement: Supplementary file 1 [file molecules-31-03372-s001.zip › molecules-4562747-supplementary.pdf]

molecules

MDPI

Supplementary Material

S1. SwissTargetPrediction Analysis of Compounds 5a–5e and 5g–5i

To complement the detailed SwissTargetPrediction analysis of compound **5f** presented in the main manuscript, target prediction was also performed for the remaining compounds **5a–5e and 5g–5i**. The distributions of the top 15 predicted targets for these compounds are presented in **Figures S1–S8**, while the ranking of EGFR across the complete series is summarized in **Table S1**.

The predicted target profiles showed considerable diversity, including kinases, erasers, hydrolases, oxidoreductases, proteases, cytochrome P450 enzymes, and other protein classes. Importantly, **epidermal growth factor receptor (EGFR; UniProt ID: P00533; ChEMBL ID: CHEMBL203)** appeared among the top 15 predicted targets for each of the eight compounds presented in the Supplementary Material. Together with the corresponding result obtained for compound **5f** in the main manuscript, EGFR was therefore identified among the top 15 predicted targets for **all nine derivatives (5a–5i)**.

The predicted EGFR ranking varied across the series, ranking **first for compounds 5a, 5g, and 5h; fifth for 5d; eighth for 5c; tenth for 5i; twelfth for 5f; thirteenth for 5b; and fourteenth for 5e** (Table S1). Thus, although its relative ranking differed among the derivatives, EGFR was recurrently identified as a **predicted target** throughout the investigated series.

Interestingly, the SwissTargetPrediction ranking did not directly parallel the molecular docking scores. Compound **5f** exhibited the most favorable docking score against EGFR (**−13.5 kcal/mol**), although EGFR ranked **twelfth** in its SwissTargetPrediction profile, whereas EGFR was the **first-ranked predicted target** for compounds **5a, 5g, and 5h**. This lack of direct correspondence indicates that the SwissTargetPrediction rankings and molecular docking scores should be regarded as **complementary computational outputs rather than directly equivalent measures**.

Overall, the recurrent identification of EGFR among the top 15 predicted targets across compounds **5a–5i** provides additional computational rationale for its investigation in the molecular docking study. Nevertheless, these predictions should be regarded as **hypothesis-generating rather than evidence of experimentally demonstrated EGFR binding or inhibition**, and appropriate **kinase-inhibition, target-engagement, and/or cellular assays** would be required for experimental validation.

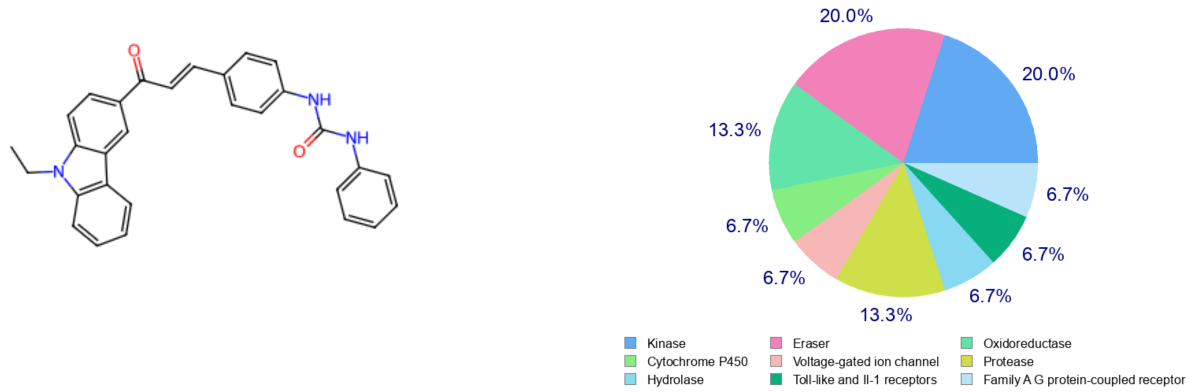

Figure S1. *SwissTargetPrediction profile of compound 5a showing the distribution of the top 15 predicted target classes.*

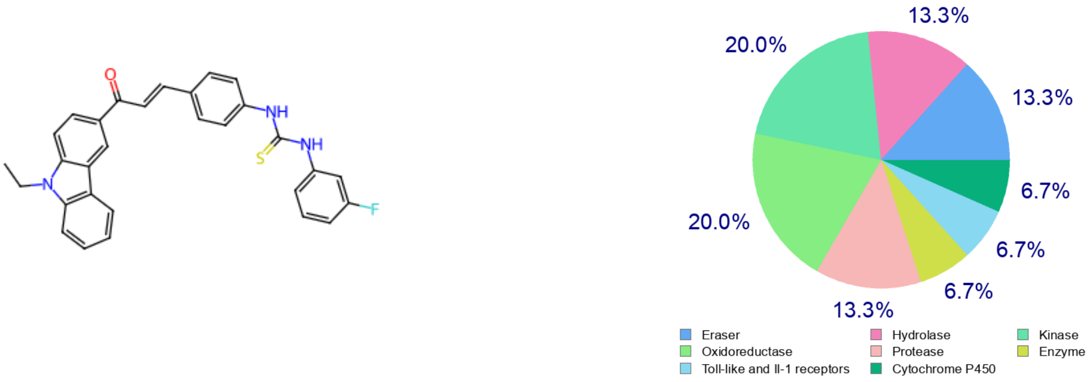

Figure S2. *SwissTargetPrediction profile of compound 5b showing the distribution of the top 15 predicted target classes.*

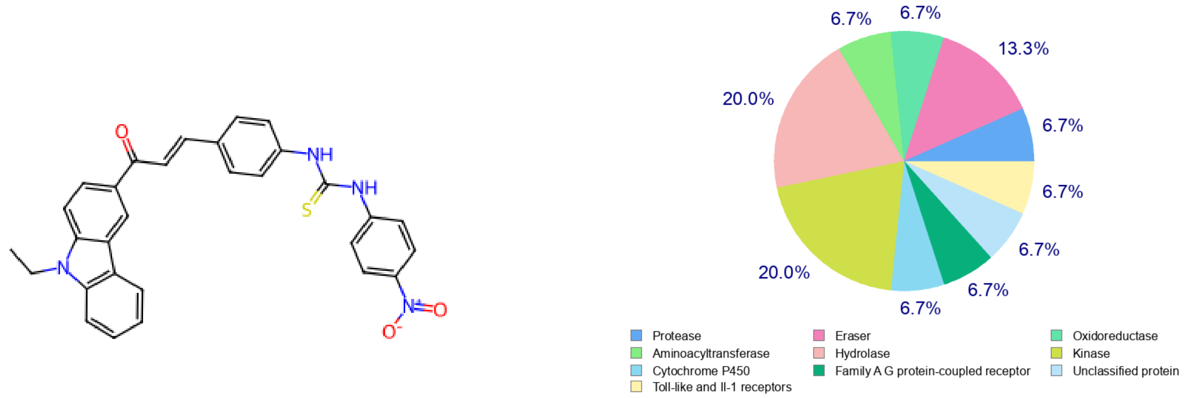

Figure S3. *SwissTargetPrediction profile of compound 5c showing the distribution of the top 15 predicted target classes.*

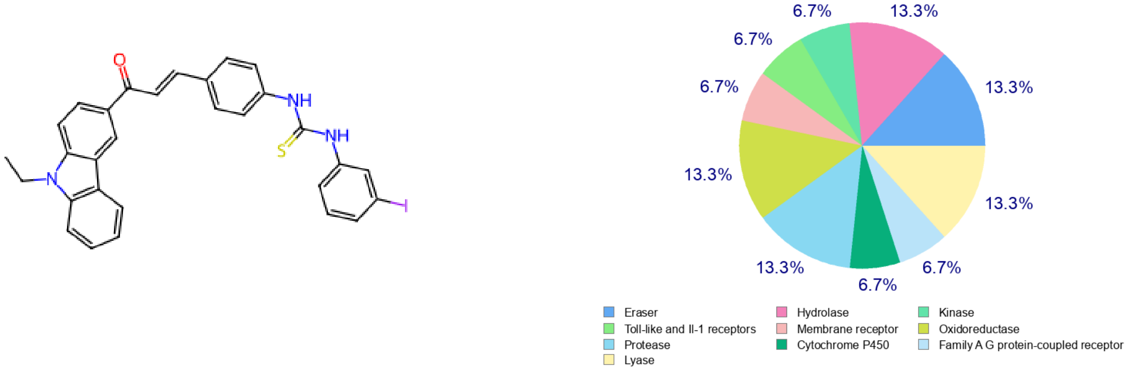

Figure S4. *SwissTargetPrediction profile of compound 5d showing the distribution of the top 15 predicted target classes.*

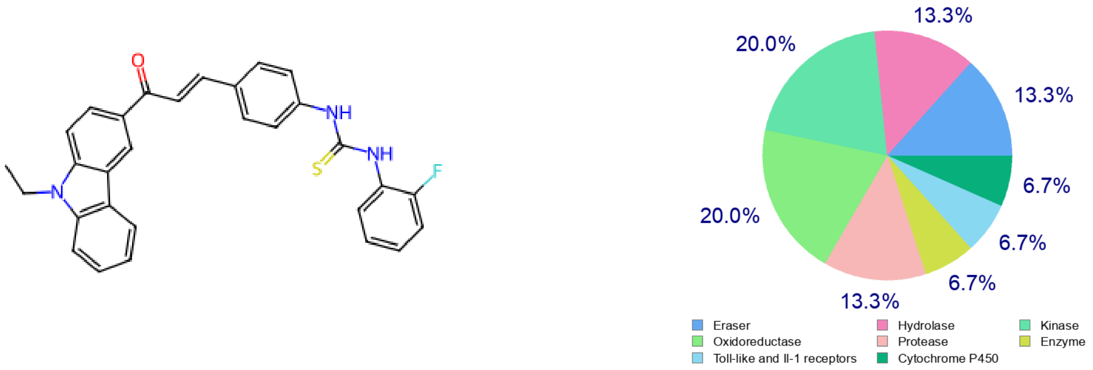

Figure S5. *SwissTargetPrediction profile of compound 5e showing the distribution of the top 15 predicted target classes.*

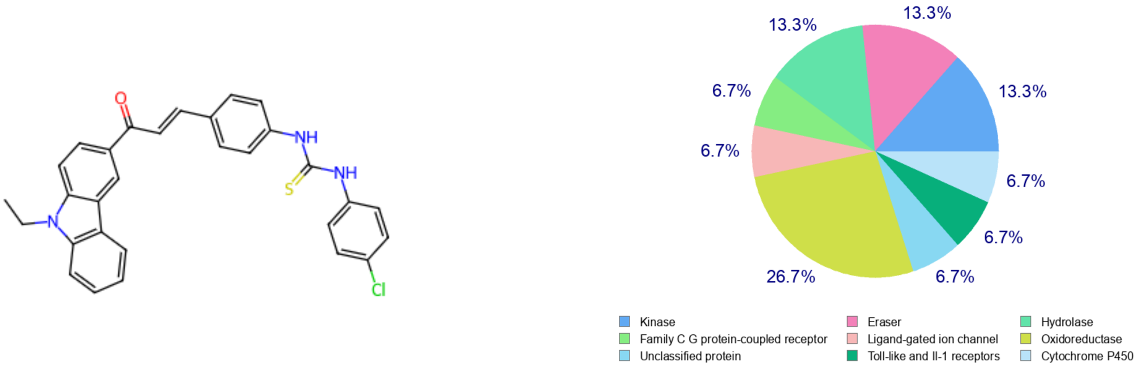

Figure S6. *SwissTargetPrediction profile of compound 5g showing the distribution of the top 15 predicted target classes.*

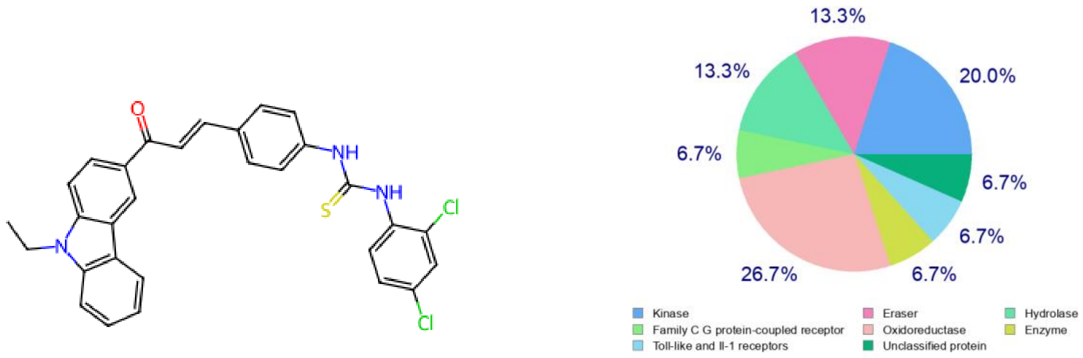

Figure S7. *SwissTargetPrediction profile of compound 5h showing the distribution of the top 15 predicted target classes.*

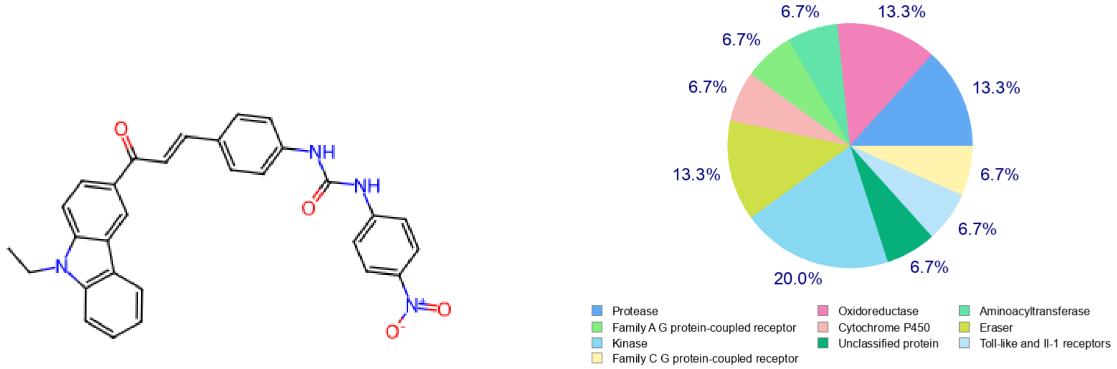

Figure S8. *SwissTargetPrediction profile of compound 5i showing the distribution of the top 15 predicted target classes.*

General description of Figures S1–S8

Figures **S1–S8** present the SwissTargetPrediction profiles of compounds **5a–5e and 5g–5i**, complementing the detailed analysis of compound **5f** provided in the main manuscript. The figures illustrate the **top 15 predicted targets** for each derivative and their associated target classes. Overall, the compounds displayed diverse predicted target profiles comprising several protein classes, including kinases, erasers, hydrolases, oxidoreductases, proteases, cytochrome P450 enzymes, and other target families. Despite variations in the identity and relative ranking of the

predicted targets among the derivatives, **kinase-related targets were recurrently represented across the series**.

Notably, **EGFR (UniProt ID: P00533; ChEMBL ID: CHEMBL203)** was identified among the top 15 predicted targets for each of the compounds presented in **Figures S1–S8**. Together with compound **5f**, whose SwissTargetPrediction profile is presented and discussed in the main manuscript, these results show that EGFR was recurrently predicted across the complete series of compounds **5a–5i**. The individual EGFR rankings for the complete series are summarized in **Table S1**. These predictions provide additional computational rationale for investigating EGFR as a potential molecular target; however, they should be regarded as **hypothesis-generating predictions rather than evidence of experimentally confirmed EGFR binding or inhibitory activity**.

Compound	EGFR predicted among Top 15	EGFR rank	UniProt ID	ChEMBL ID	Target class
5a	Yes	1	P00533	CHEMBL203	Kinase
5b	Yes	13	P00533	CHEMBL203	Kinase
5c	Yes	8	P00533	CHEMBL203	Kinase
5d	Yes	5	P00533	CHEMBL203	Kinase
5e	Yes	14	P00533	CHEMBL203	Kinase
5f*	Yes	12	P00533	CHEMBL203	Kinase
5g	Yes	1	P00533	CHEMBL203	Kinase
5h	Yes	1	P00533	CHEMBL203	Kinase
5i	Yes	10	P00533	CHEMBL203	Kinase

Table S1. *SwissTargetPrediction ranking of epidermal growth factor receptor (EGFR) among the top 15 predicted targets for compounds 5a–5i. EGFR was identified among the top 15 predicted targets for all investigated derivatives. *The detailed SwissTargetPrediction analysis of compound 5f is presented in the main manuscript.*

As summarized in **Table S1**, EGFR (UniProt ID: **P00533**; ChEMBL ID: **CHEMBL203**) was identified among the top 15 predicted targets for all nine investigated compounds, although its relative ranking varied across the series. EGFR was the first-ranked predicted target for compounds **5a, 5g, and 5h**, while it ranked fifth for **5d**, eighth for **5c**, tenth for **5i**, twelfth for **5f**, thirteenth for **5b**, and fourteenth for **5e**. The recurrent identification of EGFR across all nine derivatives indicates that this prediction was not restricted to a single compound or substituent pattern and provides additional computational rationale for investigating EGFR as a potential molecular target of this compound series.

Interestingly, the SwissTargetPrediction ranking did not directly parallel the molecular docking scores. Compound **5f** exhibited the most favorable docking score against EGFR (**−13.5 kcal/mol**), although EGFR ranked twelfth among its top 15 predicted targets. Conversely, EGFR was the first-ranked predicted target for compounds **5a, 5g, and 5h**, which did not exhibit the most favorable docking scores within the series. This lack of direct correspondence is consistent with the fact that the two computational approaches provide different types of information: molecular docking

evaluates the predicted binding pose and interaction of a ligand within a predefined receptor binding site, whereas SwissTargetPrediction provides ligand-based predictions of potential biological targets. Accordingly, the outputs of these approaches should be regarded as **complementary rather than directly comparable**.

Overall, the recurrent occurrence of EGFR among the top 15 predicted targets across the complete **5a–5i** series provides **additional computational rationale for its selection as the receptor investigated in the molecular docking analysis**. Nevertheless, these results do not demonstrate direct EGFR binding or inhibition and should therefore be interpreted as **hypothesis-generating predictions**. Experimental **EGFR kinase-inhibition, target-engagement, and/or cellular assays** would be required to establish EGFR-related biological activity.

S2. Two-Dimensional Docking Interaction Analysis

To complement the detailed binding-mode analysis of compound **5f** presented in the main manuscript, two-dimensional interaction diagrams were also generated for the remaining compounds **5a–5e and 5g–5i** within the **EGFR binding pocket (PDB ID: 1M17)**. The corresponding interaction maps are presented in **Figures S9–S16** and illustrate the predicted hydrogen-bonding, hydrophobic, π-related, halogen, and other non-covalent contacts between the ligands and surrounding amino acid residues.

These supplementary interaction diagrams provide a broader representation of the **predicted protein–ligand interaction patterns** across the investigated series and facilitate comparison with compound **5f**. As these interactions were derived from molecular docking calculations, they should be interpreted as **computationally predicted binding contacts rather than experimentally confirmed ligand–receptor interactions**.

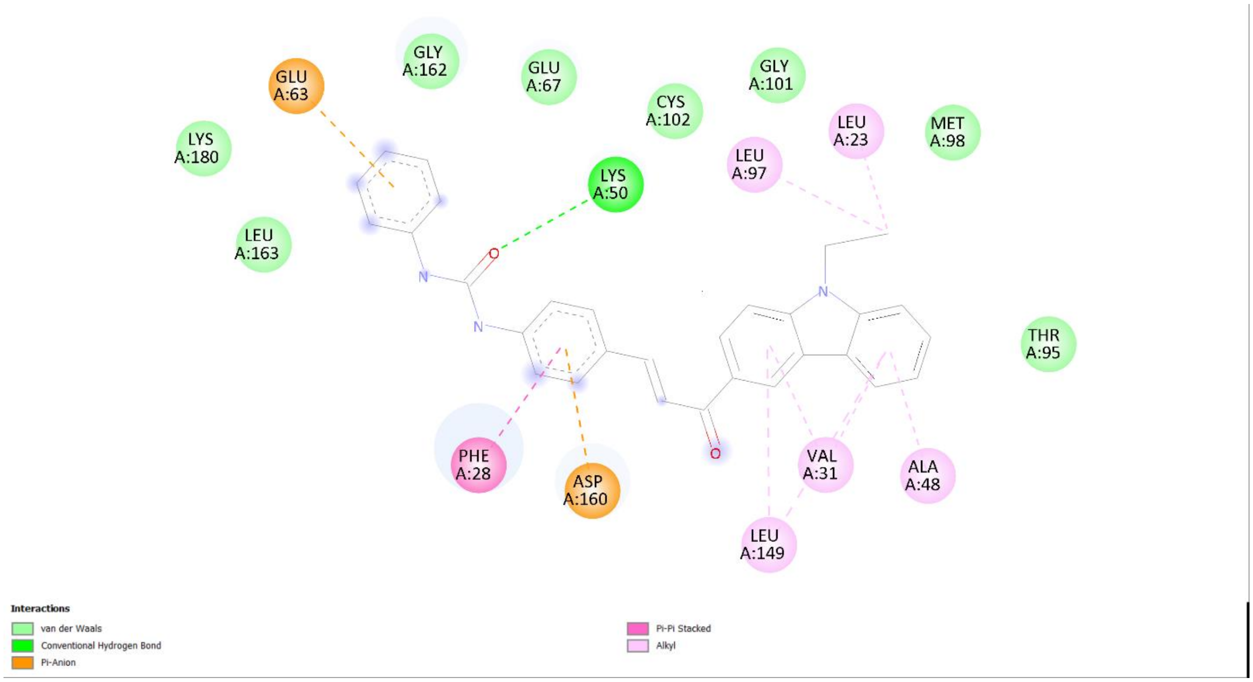

Figure S9. *Two-dimensional interaction diagram of compound 5a within the predicted binding pocket of EGFR tyrosine kinase (PDB ID: 1M17), showing the principal predicted hydrogen-bonding, π-related, hydrophobic, and van der Waals interactions with surrounding amino acid residues.*

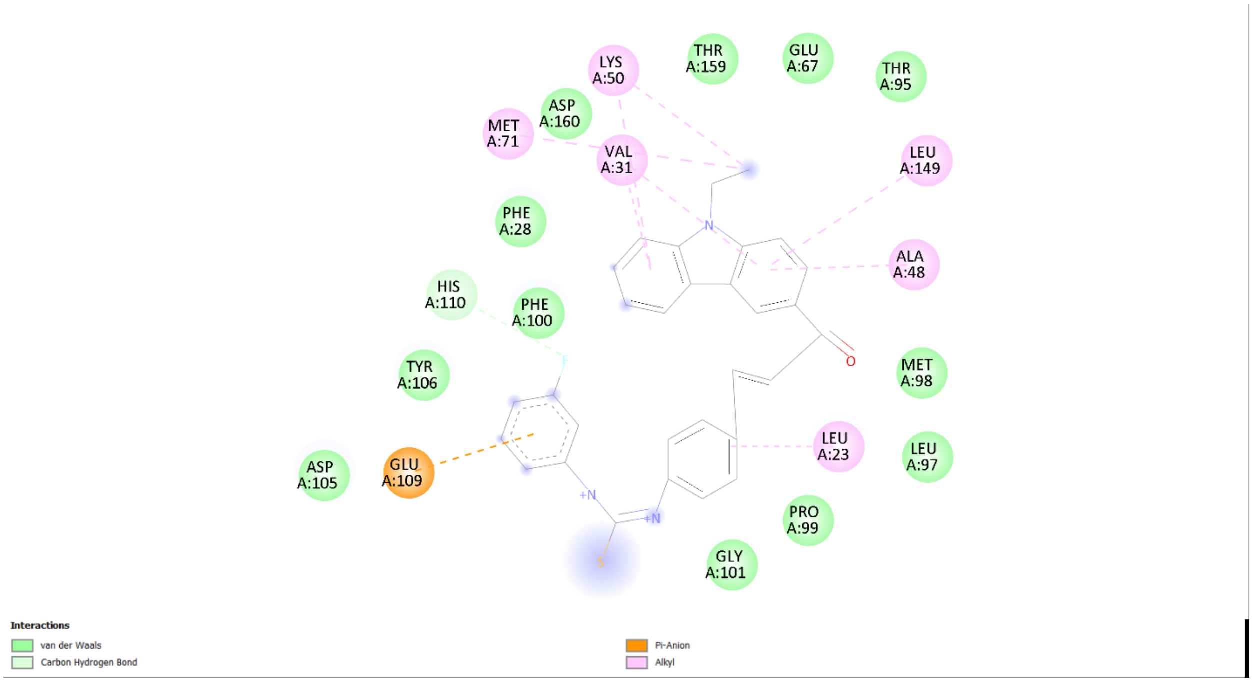

Figure S10. *Two-dimensional interaction diagram of compound 5b within the binding pocket of EGFR (PDB ID: 1M17), showing the predicted ligand–residue interactions obtained from the molecular docking analysis.*

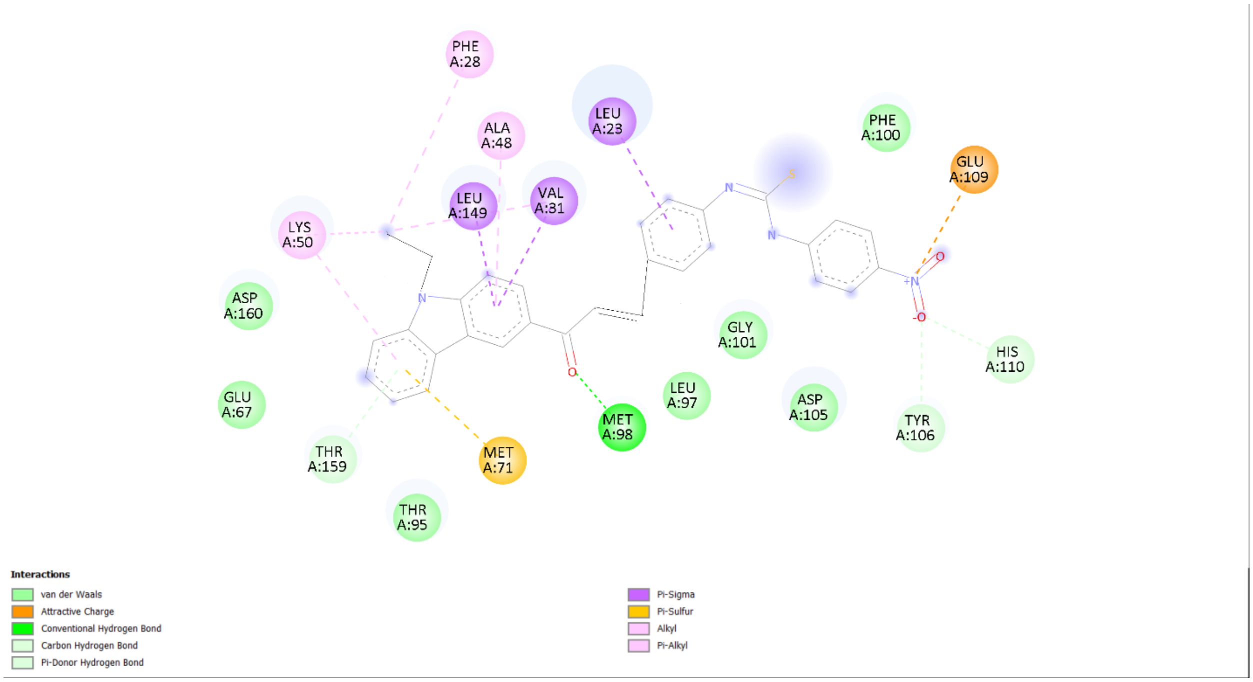

Figure S11. *Two-dimensional interaction diagram of compound 5c within the binding pocket of EGFR (PDB ID: 1M17), showing the predicted ligand–residue interactions obtained from the molecular docking analysis.*

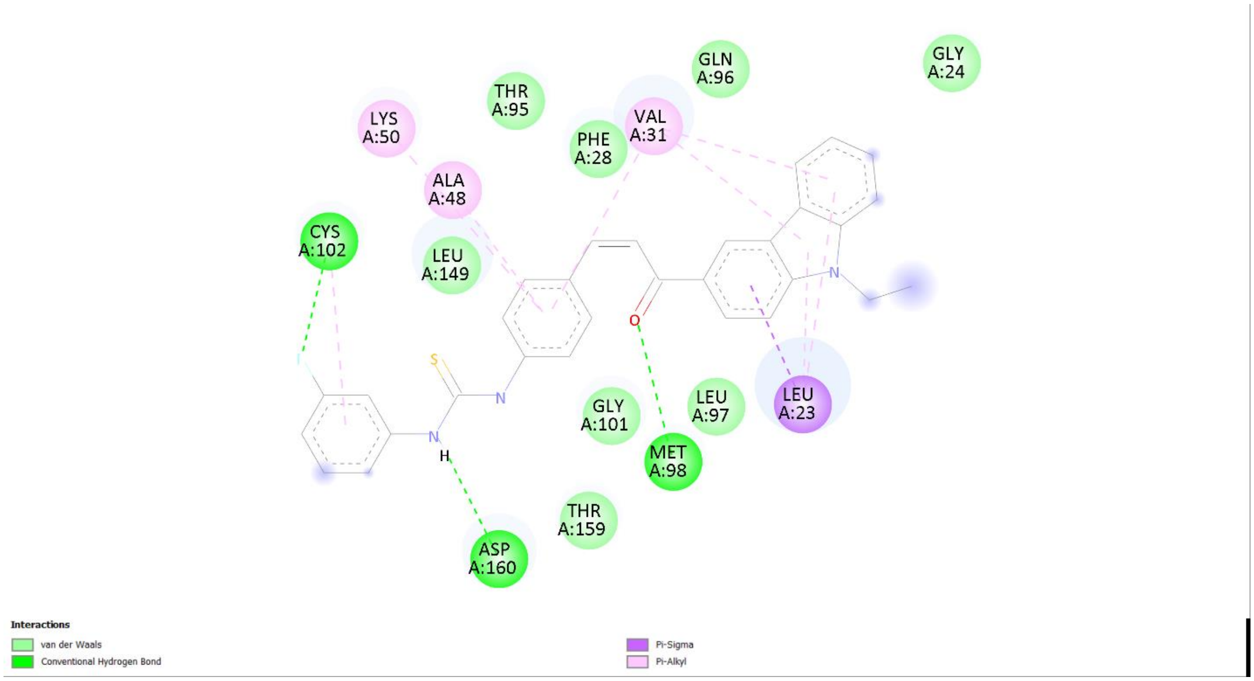

Figure S12. *Two-dimensional interaction diagram of compound 5d within the binding pocket of EGFR (PDB ID: 1M17), showing the predicted ligand–residue interactions obtained from the molecular docking analysis.*

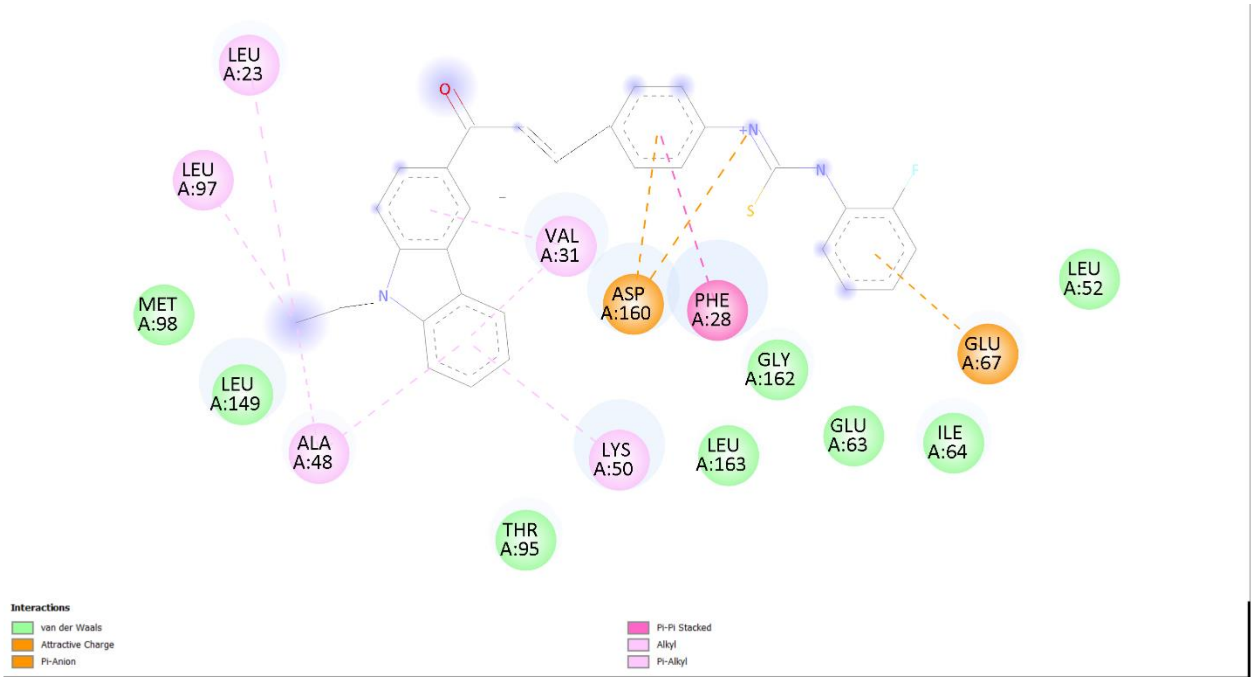

Figure S13. *Two-dimensional interaction diagram of compound 5e within the binding pocket of EGFR (PDB ID: 1M17), showing the predicted ligand–residue interactions obtained from the molecular docking analysis.*

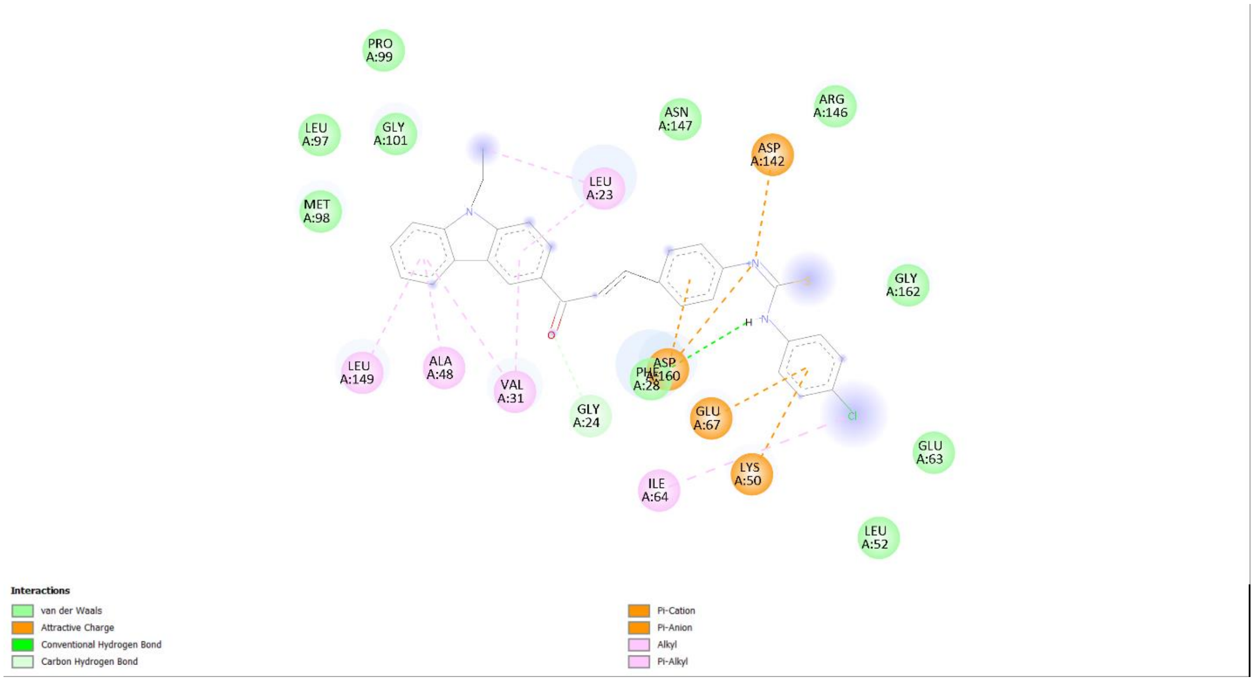

Figure S14. *Two-dimensional interaction diagram of compound 5g within the binding pocket of EGFR (PDB ID: 1M17), showing the predicted ligand–residue interactions obtained from the molecular docking analysis.*
